# Comparative efficacy and safety of catheter ablation technologies for atrial fibrillation: a systematic review and network meta-analysis

**DOI:** 10.3389/fcvm.2026.1667194

**Published:** 2026-04-14

**Authors:** Ruiting Feng, Jia Gao, Yajie Guo, Yuli Guo, Rui Wang, Min Guo

**Affiliations:** 1 Department of Cardiology, First Hospital of Shanxi Medical University, Taiyuan, Shanxi, China; 2 The First Clinical Medical College, Shanxi Medical University, Taiyuan, Shanxi, China

**Keywords:** atrial fibrillation, cryoballoon ablation, network meta-nanlysis, pulsed field ablation, radiofrequency ablation, remote magneticnavigation ablation

## Abstract

**Aims:**

There are several interventional approaches to atrial fibrillation (AF) ablation; however, it is not yet known which procedure is most effective. Our aim was to compare the efficacy and safety of different interventional approaches for the treatment of AF through network meta-analysis.

**Methods:**

We searched randomized controlled trial (RCT) and propensity-score matched (PSM) studies in PubMed, Embase, and Cochrane Library databases from the initial period to December 2024, and studies were selected which had cryoballoon ablation (CBA), conventional radiofrequency ablation (RFA), remote magnetic navigation ablation (RMN), and pulsed field ablation (PFA) as an arm in the study. Network meta-analysis (NMA) was performed using a frequentist approach with STATA (version 14.0) software.

**Results:**

We included 10 RCT studies and 4 PSM studies. For freedom from AF and other atrial tachyarrhythmias (AT) indicators, PFA may become the most effective ablation procedure (SUCRA = 88.4%, RR = 1.11, 95% CI: 1.03–1.21). For procedure duration, PFA may also be the ablation procedure with the best results (SUCRA = 91.2%, SMD = −1.43, 95% CI: −2.47 to −0.39).

**Conclusion:**

The choice of ablation technique needs to be weighed against the specific clinical needs and the patient's situation. PFA may be the best choice if success rate and procedure time are prioritized, while RMN is more appropriate if complication rates and fluoroscopy time are more important.

**Systematic Review Registration:**

https://www.crd.york.ac.uk/prospero/display_record.php?ID=CRD42025631158, identifier: CRD42025631158.

## 1 Introduction

Atrial fibrillation (AF) is the most common chronic arrhythmia, increasing the risk of death, congestive heart failure (CHF), and embolic phenomena (1). Catheter ablation is an established treatment option for patients with symptomatic atrial fibrillation, resistant to anti-arrhythmic medications (2). Circumferential pulmonary vein isolation (PVI) is the mainstay of AF ablation. Traditionally, pulmonary vein isolation by radiofrequency ablation has been achieved using a point-by-point technique and 3D navigation, which makes it difficult to create round and contiguous lesions, and conduction gaps may occur in the presence of poor catheter-tissue contact and incomplete non-permeable lesions; this approach may also be hampered by a long learning curve (3). To overcome these problems, new ablation techniques are constantly being developed to improve the efficacy and safety of AF ablation.

Cryoballoon ablation (CBA) offers several advantages, including a rapid learning curve and shorter procedure times. Considering the optimized catheter design, it has been shown that a single cryo-per-vein strategy provides favorable safety and clinical outcomes, questioning the need for routine “extra cryo” applications. In addition, shorter freezing times and dose-adjustment strategies based on multiparametric assessment may further simplify the approach to freezing AF and reduce the risk of complications by ensuring successful long-term outcomes (3). Although cryoballoon was introduced as an alternative method to achieve complete pulmonary vein isolation, it is also associated with a significant risk of phrenic nerve and esophageal injury and has no real advantage in terms of arrhythmia recurrence (4).

Since the first use of remote magnetic navigation (RMN) systems for cardiac ablation in 2003, its use has increased significantly due to the presence of four unique features and benefits: (1) increased precision in catheter movement and control, (2) increased catheter stability with sustained contact with tissues, (3) reduced risk of cardiac perforation due to catheter compliance, and (4) reduced exposure to x-ray fluoroscopy for both patients and physicians. Yet how and whether these potential benefits translate into short-, intermediate-, and long-term clinical outcomes remains to be determined (5).

Pulsed field ablation (PFA) is a new energy source through which high-voltage electrical pulses are used to create holes in cell membranes (i.e., electroporation), leading to cell death. The energy required to produce irreversible electroporation is highly tissue-dependent. Current clinical evidence has demonstrated significant efficacy in achieving durable pulmonary vein isolation without ablation-related adverse events. PFA represents a significant advancement in the field of catheter ablation (CA), with an expected superior safety profile compared with RFA, but further studies are needed to determine the long-term efficacy and safety of this novel energy source (6).

Given the diversity of forms and fragmentation of research in the field of AF ablation therapy, there is an urgent need for a comprehensive synthesis of the topic using evidence from high-quality trials. This can be addressed through network meta-analysis (NMA), which is an effective way to pool effect sizes across multiple studies and models. Because of the limited number of randomized controlled trial (RCT) in this area, propensity-score matched studies (PSM) were also sought to pool data. PSM have been shown to be empirically equivalent to RCT in generating unbiased estimates of the efficacy of treatment, while eliminating confounding factors and biases to a large extent (7). Therefore, the purpose of this NMA was to evaluate the efficacy and safety of various ablation therapies in patients with AF in RCT or PSM.

## 2 Methods

The investigation was conducted in accordance with the principles and guidelines of the Preferred Reporting Items for Systematic Reviews and Meta-Analyses of Network Meta-Analyses (PRISMA-NMA) (8). This systematic review was registered with PROSPERO (CRD42025631158).

### 2.1 Search strategy

An electronic literature search of relevant articles was conducted from inception through December 2024 in databases such as Embase, Cochrane Library, and PubMed, with no language restrictions. We used Medical Subject Headings (MeSH) terms, title, abstract, and related terms in our search strategy, including “atrial fibrillation”, “radiofrequency ablation”, “cryoballoon ablation”, “magnetic navigation ablation”, and “pulsed field ablation”. Appendix 1 S1 presents the search strategy details.

### 2.2 Inclusion and exclusion criteria

Inclusion criteria: (1) population: patients with AF (either paroxysmal or persistent) over 18 years of age who did not undergo any type of ablation procedure; (2) intervention: CBA, RMN, or PFA; (3) comparison: RFA or one of the CBA, RMN and PFA; (4) outcome: studies providing at least one relevant outcome, including freedom from AF or other atrial tachyarrhythmias (AT), complications, procedure duration, and fluoroscopy duration (5); study design: the type of trial was RCT or PSM.

Exclusion criteria: (1) overlapping patient populations; (2) patients who had previously undergone catheter ablation for atrial fibrillation; (3) unclear study design or subgroups; (4) no control group; (5) studies having data that are missing, incomplete, or contain missing data points; (6) conference abstracts, case reports, case series, editorials, and review articles.

### 2.3 Study selection and data extraction

First, two authors independently reviewed the literature for titles and abstracts based on the search strategy, inclusion criteria, and exclusion criteria. Second, after the initial screening, an in-depth full-text review of the remaining literature was conducted. Finally, the two authors extracted data separately. When disagreements arose, a third author was consulted to help reach agreement. Specific data extracted included authors' names, year, study design, sample size, age and sex proportions, proportion of AF types, interventions, blanking periods, and follow-up times. Where standard deviations (SDs) were not provided, they were derived from standard errors (SEs), confidence intervals (CIs), or t or *p* values, or attempts were made to contact the authors at least three times through e-mails to collect the missing data. GetData Digitizer version 2.20 software was used to extract the data we required from graphs when the authors did not provide the data in the study but instead provided a graph containing the data.

### 2.4 Bias assessment and quality of evidence

The Newcastle-Ottawa Scale (NOS) was used for quality assessment of PSM (9). The scale consists of three parts, namely, selection, comparability, and outcome, with a total of eight entries. The semi-quantitative principle of the star system was used to evaluate the quality of the literature, except for comparability, which could be assessed with a maximum of 2 stars, and the rest of the entries could be assessed with a maximum of 1 star, out of a total of 9 stars, with higher scores suggesting a higher quality of the study. Quality assessment of RCT was performed using the Cochrane Collaboration Network's Risk of Bias Assessment Tool for Randomized Trials (10). The Cochrane Risk of Bias Assessment Tool assesses the risk of bias from six domains, namely, selection bias, performance bias, detection bias, attrition bias, reporting bias, and other biases, as well as seven evaluation items (selection bias includes two evaluation items). For each evaluation item, the criteria of “low risk”, “unclear”, or “high risk” were used, depending on the circumstances.

### 2.5 Statistical analysis

Traditional meta-analysis was employed to contrast the efficacy and safety of various ablation strategies. To minimize errors, we calculated risk ratio (RR), standardized mean differences (SMDs) and 95% confidence intervals (CIs). Statistical heterogeneity is measured with the I² statistic. If I² exceeds 50%, it is considered to be significantly heterogeneity (11). If significant heterogeneity exists, use random-effects model; otherwise, use fixed-effects model (12).

This network meta-analysis was carried out in line with the PRISMA-NMA recommendations (13). We utilized a frequentist approach in conjunction with STATA (version 14.0) software for data analysis, with the aim of evaluating the relative treatment effects. This network meta-analysis integrates direct evidence derived from head-to-head comparisons within RCT and PSM along with indirect evidence. The “Network” software package was utilized for data analysis and the creation of a visual network structure graph. In this graph, each node symbolizes a distinct intervention. The magnitude of the nodes corresponds to the quantity of participants involved in various ablation strategies. When there is a straight line linking two nodes, it signifies that a direct comparison between the respective strategies was carried out in the studies that were included in the analysis. Moreover, the thickness of each straight line is in direct proportion to the number of studies that conducted a comparison between the two pertinent ablation strategies (14).

In cases where direct and indirect evidence present discrepant results for the identical treatment effect, inconsistency is gauged through the application of a node-splittong approach (15). This method sets direct evidence, which is sourced from paired trials, in contrast to indirect evidence that obtained from network meta-analyses. When the evidence is “split” at particular critical points, one can evaluate the compatibility between the direct and indirect evidence (16, 17). At every node within the network, pairwise comparisons were carried out, and the *p*-value related to each comparison was evaluated. If a *p*-value exceeds 0.05, it suggests that the direct and indirect evidence show consistency, meaning there is no statistically notable inconsistency. Conversely, when a *p*-value is less than or equal to 0.05, it signals potential inconsistency. The rank of intervention was accomplished by us using the surface under the cumulative ranking curve (SUCRA) (18). In the end, publication bias was verified by creating a funnel plot and conducting a symmetry criterion check (14).

## 3 Results

### 3.1 Study selection

**Figure 1:**
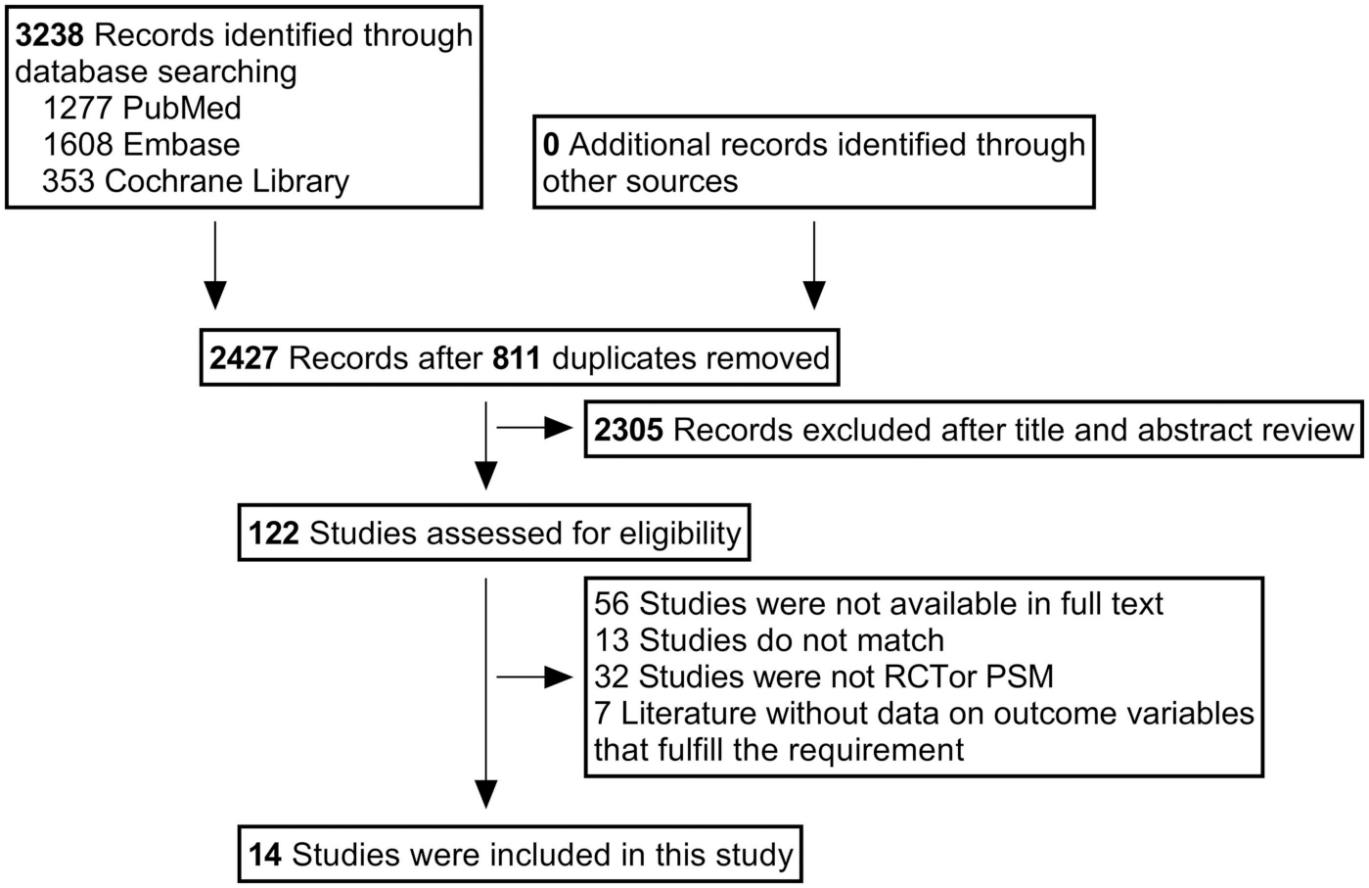
PRISMA flow diagram.

3,238 studies were identified based on the search strategy. Firstly, 811 duplicate studies were removed and secondly 2,305 studies were screened by reading titles and abstracts. Fourteen studies (10 RCT and 4 PSM) were included in this network meta-analysis through inclusion and exclusion criteria, including 4,055 patients (Figure 1).

### 3.2 Study characteristics

Of these studies, 11 compared CBA with RFA (4, 19–28), 1 compared RMN with RFA (29), and the remaining 2 compared CBA, PFA, and RFA (30, 31). The majority of the studies did not report the type of AF, and the male-to-female ratio was approximately 2:1. A 90-day blanking period was implemented in virtually all of the studies, in which recurrences of AF or atrial tachycardia after the first ablation were not counted as a failure of therapy. Follow-up was greater than or equal to 12 months (Table 1).

**Table 1:** Characteristics of included study.

Study	Design	Comparator arms	Study size	Age, year	Female/male	PAF/PerAF	Blanking period, days	Follow-up duration, months
Pulido Adragão et al, 2016	PSM	RFA vs. RMN	287 vs. 287	57.9 ± 11.2 vs. 58.3 ± 11.7	93/194 vs. 86/201	207/80 vs. 213/74	30	≥18
Andrade et al, 2019	RCT	RFA vs. CBA	115 vs. 115	58.6 ± 9.2 vs. 59.6 ± 9.9	36/79 vs. 34/81	105/10 vs. 109/6	30	12
Andrade et al, 2024	RCT	RFA vs. CBA	115 vs. 115	60.0 ± 10.4 vs. 61.0 ± 9.6	36/79 vs. 34/81	115/0 vs. 115/0	30	≥18
Baimbetov et al, 2022	RCT	RFA vs. CBA	50 vs. 50	61.6 ± 6.5 vs. 61.3 ± 10.2	21/29 vs. 19/31	0/50 vs. 0/50	30	≥18
Davtyan et al, 2018	RCT	RFA vs. CBA	44 vs. 45	55.6 ± 12.0 vs. 57.6 ± 8.2	25/19 vs. 23/22	44/0 vs. 45/0	30	12
Della Rocca et al, 2023	PSM	RFA vs. CBA vs. PFA	348 vs. 348 vs. 174	62.9 ± 10.1 vs. 62.1 ± 12.3 vs. 62.0 ± 11.6	131/217 vs. 130/218 vs. 64/110	348/0 vs. 348/0 vs. 174/0	30	12
Hunter et al, 2015	RCT	RFA vs. CBA	77 vs. 78	61.0 ± 12.0 vs. 56.0 ± 11.0	30/47 vs. 22/56	77/0 vs. 78/0	30	12
Kuck et al, 2016	RCT	RFA vs. CBA	376 vs. 374	60.1 ± 9.2 vs. 59.9 ± 9.8	140/236 vs. 153/221	376/0 vs. 374/0	30	≥18
Luik et al, 2015	RCT	RFA vs. CBA	159 vs. 156	60.0 ± 9.6 vs. 61.0 ± 8.9	68/91 vs. 56/100	159/0 vs. 156/0	30	12
Matta et al, 2018	PSM	RFA vs. CBA	46 vs. 46	59.0 ± 9.0 vs. 59.0 ± 9.0	8/38 vs. 10/36	46/0 vs. 46/0	30	12
Maurhofer et al, 2024	PSM	RFA vs. CBA vs. PFA	80 vs. 80 vs. 40	62.0 ± 10.6 vs. 62.2 ± 11.9 vs. 62.6 ± 9.1	17/63 vs. 22/58 vs. 10/30	80/0 vs. 80/0 vs. 80/0	30	12
Mililis et al, 2023	RCT	RFA vs. CBA	133 vs. 66	60.2 ± 9.9 vs. 62.7 ± 9.1	22/111 vs. 15/51	0/133 vs. 0/66	30	12
Shi et al, 2022	RCT	RFA vs. CBA	50 vs. 51	64.0 ± 8.7 vs. 62.4 ± 8.4	15/35 vs. 6/45	0/50 vs. 0/51	30	12
Theis et al, 2022	RCT	RFA vs. CBA	75 vs. 75	N/A	N/A	75/0 vs. 75/0	NA	15

PAF, paroxysmal atrial fibrillation; PerAF, persistent atrial fibrillation; N/A, not applicable.

### 3.3 Risk of bias assessment

The Newcastle-Ottawa Scale (NOS) was used for quality assessment of PSM studies (Appendix 1, S2, Supplementary Table S1). Quality assessment of RCT was performed using the Cochrane Collaboration Network's Risk of Bias Assessment Tool for Randomized Trials (Appendix 1, S2, Figure S1). The majority of the studies showed proper randomization and comparable baselines, indicating that the groups were well-matched at the onset of the trial. Nevertheless, given the characteristics of the interventions, implementing blinding was especially arduous. It was tough to keep physicians, patients, and outcome assessors in the dark. This absence of blinding might bring about biases, potentially undermining the reliability of the results.

### 3.4 Outcome-freedom from AF and other AT

**Figure 2:**
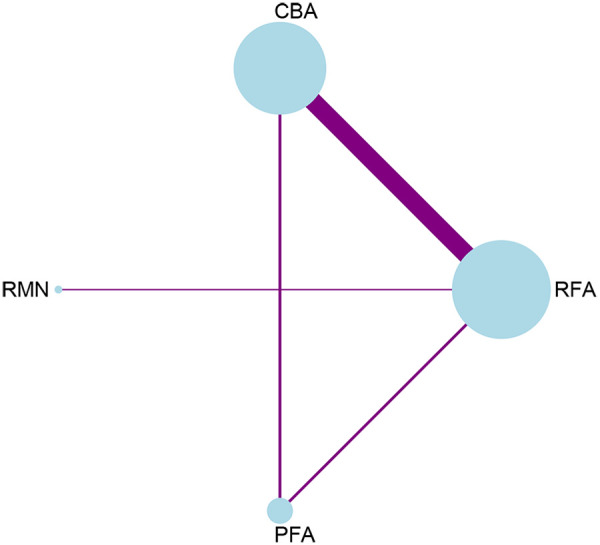
Network plot for the outcome of freedom from AF and other AT.

**Figure 3:**
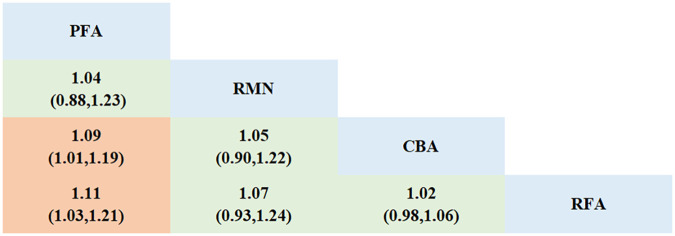
League table for the outcome of freedom from AF and other AT.

14 studies that focused on freedom from AF and other AT, and involved 4 interventions, were incorporated. Every ablation procedure was compared directly to RFA (Figure 2, Appendix 1, S3, Figure S2). However, studies directly comparing CBA, PFA with RMN are scarce. In the inconsistency test analysis for the outcome of “freedom from AF and other AT”, no significant heterogeneity was detected (*P* = 0.8473 > 0.05) (Appendix 1, S3, Figure S3). There was also no statistically significant inconsistency between the direct and indirect evidence among different ablation modalities (RFA vs. PFA, *p* = 0.847 > 0.05; CBA vs. PFA, *p* = 0.847 > 0.05). Through network meta-analysis, our study demonstrated that compared with RFA, PFA [RR = 1.11, 95% CI (1.03, 1.21)] showed a lower recurrence rate of AF and other AT. Additionally, the efficacy of PFA [RR = 1.09, 95% CI (1.01, 1.19)] was superior to that of CBA. No significant differences were observed between the remaining intervention modalities (Figure 3).

The Surface Under the Cumulative Ranking Curve (SUCRA) values reflected the efficacy ranking of different ablation modalities (Appendix 1, S3, Figure S4). Among the four ablation modalities, PFA had the highest probability of being the optimal intervention (SUCRA = 88.4%, PrBest = 67.0%, MeanRank = 1.3); followed by the suboptimal intervention, namely RMN (SUCRA = 63.6%, PrBest = 32.3%, MeanRank = 2.1); CBA came next (SUCRA = 35.9%, PrBest = 0.6%, MeanRank = 2.9); and RFA had the lowest SUCRA value (SUCRA = 12.1%, PrBest = 0.1%, MeanRank = 3.6). The comparison-adjusted funnel plot provided no evidence of significant publication bias (Appendix 1, S3, Figure S6).

### 3.5 Outcome-complications

**Figure 4:**
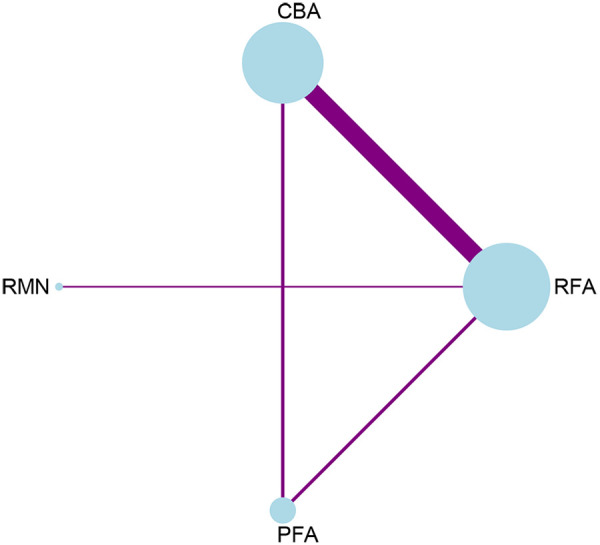
Network plot for the outcome of complications.

**Figure 5:**
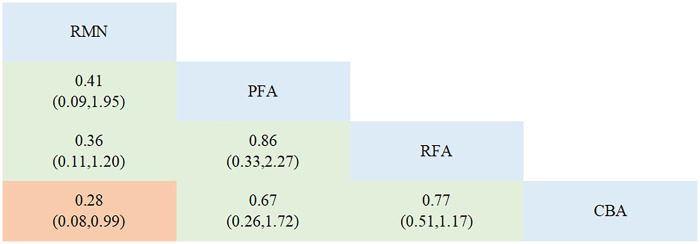
League table for the outcome of complications.

Regarding complications, 12 studies that involved 4 interventions were taken into account. Each ablation procedure underwent a direct comparison with RFA (Figure 4, Appendix 1, S4, Figure S7). In the inconsistency analysis of the complication outcome, no notable heterogeneity was identified (*P* = 0.6878 > 0.05) (Appendix 1, S4, Figure S8). Similarly, no statistically significant inconsistency was observed between the direct and indirect evidence across different ablation techniques: the *p*-values for the comparisons of RFA vs. PFA and CBA vs. PFA were both 0.688, which were greater than 0.05. Results from the network meta-analysis indicated that, in comparison to RFA, RMN and PFA tended to have lower complication rates, while CBA had the highest complication rate. Nevertheless, these differences did not reach statistical significance. The only conclusive result was that RMN [RR = 0.28, 95% CI (0.08, 0.99)] was associated with a significantly lower complication rate than CBA (Figure 5).

The SUCRA value reflects the safety ranking of different ablation modalities. (Appendix 1, S4, Figure S9). RMN had the greatest likelihood of being the optimal intervention (SUCRA = 92.9%, PrBest = 85.0%, MeanRank = 1.2). It was followed by PFA, which was the second-best intervention (SUCRA = 51.9%, PrBest = 12.9%, MeanRank = 2.4). RFA came next in the ranking (SUCRA = 43.9%, PrBest = 1.9%, MeanRank = 2.7), and CBA had the lowest SUCRA value (SUCRA = 11.3%, PrBest = 0.2%, MeanRank = 3.7). No evidence of significant publication bias was found in the comparison-adjusted funnel plot (Appendix 1, S4, Figure S11).

### 3.6 Outcome-procedure duration

**Figure 6:**
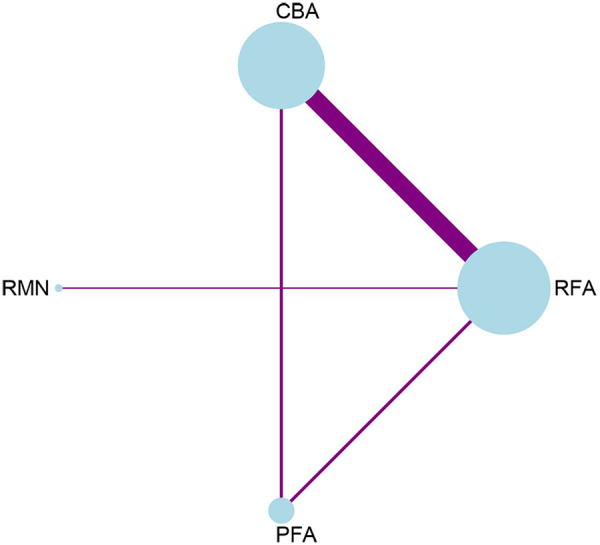
Network plot for the outcome of procedure duration.

**Figure 7:**
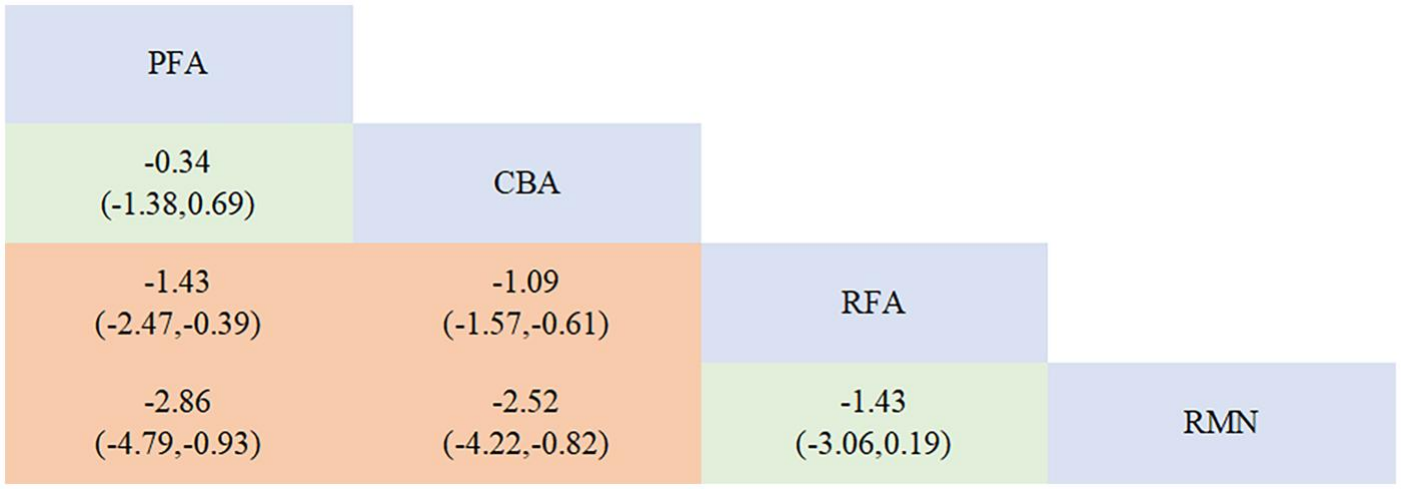
League table for the outcome of procedure duration.

A direct comparison was made between each ablation procedure and RFA (Figure 6). Moreover, direct comparisons among the various ablation procedures were conducted (Appendix 1, S5, Figure S12). When performing inconsistency detection in network analysis, no substantial inconsistencies were found (*P* = 0.3606 > 0.05) (Appendix 1, S5, Figure S13). No statistically significant inconsistency was detected between direct and indirect evidence across the ablation techniques (RFA vs. PFA, *p* = 0.360 > 0.05; CBA vs. PFA, *p* = 0.360 > 0.05). Through network meta-analysis, our study demonstrated that the procedure duration of PFA [SMD = −1.43, 95% CI (−2.47, −0.39)] and CBA [SMD = −1.09, 95% CI (−1.57, −0.61)] was significantly shorter than that of RFA. However, RMN had the longest procedurr duration. Specifically, the procedure duration of PFA [SMD = −2.86, 95% CI (−4.79, −0.93)] and CBA [SMD = −2.52, 95% CI (−4.22, −0.82)] was shorter than that of RMN, while no statistically significant difference in procedure duration was observed between RFA and RMN (Figure 7).

The ranking of diverse ablation procedures is mirrored by SUCRA (Appendix 1, S5, Figure S14). PFA was the safest ablation modality (SUCRA = 91.2%, PrBest = 73.9%, MeanRank = 1.3); CBA ranked second (SUCRA = 75.3%, PrBest = 26.0%, MeanRank = 1.7); RFA followed next (SUCRA = 32.0%, PrBest = 0.0%, MeanRank = 3.0); and RMN had the lowest SUCRA value (SUCRA = 1.5%, PrBest = 0.1%, MeanRank = 4.0). The funnel plot did not show any significant signs of asymmetry (Appendix 1, S5, Figure S16).

### 3.7 Outcome-fluoroscopy duration

**Figure 8:**
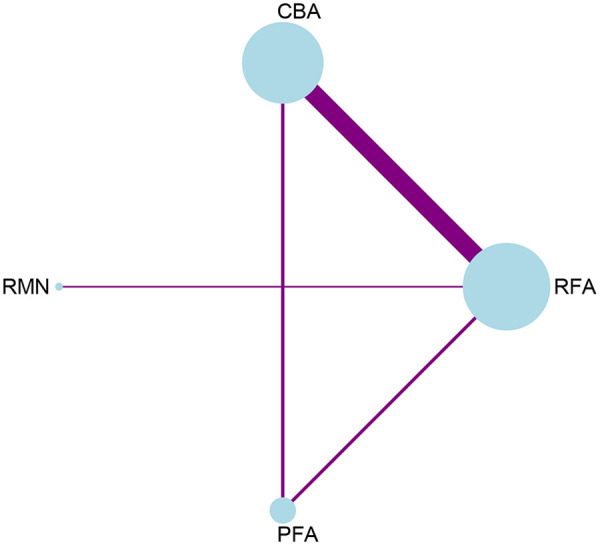
Network plot for the outcome of fluoroscopy duration.

**Figure 9:**
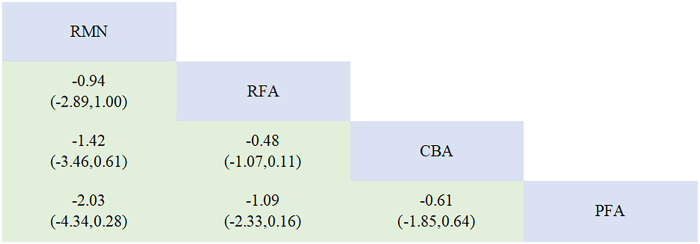
League table for the outcome of fluoroscopy duration.

Direct comparison were made between RFA and different ablation procedures (Figure 8, Appendix 1, S6, Figure S17). Following the network analysis, the result of the inconsistency test showed no substantial signs of inconsistency (*P* = 0.4078 > 0.05) (Appendix 1, S6, Figure S18). No statistically significant inconsistency was detected between direct and indirect evidence across the ablation techniques (RFA vs. PFA, *p* = 0.408 > 0.05; CBA vs. PFA, *p* = 0.408 > 0.05). Unfortunately, the results of the network meta-analysis for the four ablation modalities did not show any significant statistical differences (Figure 9). SUCRA reveals the ranking of various ablation procedures (Appendix 1, S6, Figure S19), RMN was identified as the safest ablation technique (SUCRA = 89.4%, PrBest = 81.5%, MeanRank = 1.3), RFA ranked second (SUCRA = 69.3%, PrBest = 16.1%, MeanRank = 1.9), followed by CBA (SUCRA = 32.5%, PrBest = 1.1%, MeanRank = 3.0), while PFA showed the lowest SUCRA value (SUCRA = 8.8%, PrBest = 1.3%, MeanRank = 3.7). There was no evidence of significant asymmetry in the funnel plot (Appendix 1, S6, Figure S21).

### 3.8 Subgroup analysis and sensitivity analysis

Based on the analysis results of the primary outcome (freedom from AF and other AT), subgroup analyses were conducted separately for paroxysmal atrial fibrillation, persistent atrial fibrillation, as well as for RCT and PSM. A consistent trend was observed in most subgroups. For paroxysmal atrial fibrillation, PFA [RR = 1.12, 95% CI (1.03, 1.21)] remained superior to RFA. In the PSM subgroup, PFA [RR = 1.11, 95% CI (1.02,1.21)] also showed the same favorable result (Appendix 1, S7, Figures S22–S25). Regarding secondary outcomes (complications, procedure duration, and fluoroscopy duration), corresponding subgroup analyses were performed, and the same trend was still observed (Appendix 1, S7, Figures S26–S37).

Based on the primary outcome of freedom from AF and other AT, we found that after excluding trials with a follow-up duration longer than 12 months, the results of the sensitivity analysis were consistent with the previous findings. PFA [RR = 1.11, 95% CI (1.03, 1.21)] was the most effective ablation modality, followed by CBA [RR = 1.02, 95% CI (0.96, 1.08)] (Appendix 1, S8, Figures S38–S41). In addition, corresponding sensitivity analyses were conducted based on the secondary outcomes (complications, procedure duration, and fluoroscopy duration), and all results were consistent with the previous ones (Appendix 1, S8, Figures S42–S53).

## 4 Discussion

Since the identification of the focal origin of atrial ectopic beats and their successful ablation using radiofrequency energy, the follow-up model aims to achieve the same results with different methods of energy delivery (32). With advances in technology, ablation is becoming safer and more effective (33). Thus, meta-analyses of the efficacy and safety of CBA vs. RFA or RMN vs. RFA have been performed previously (34, 35). However, this can only provide a fragmented view of the larger picture of AF ablation therapy. This network meta-analysis compared the efficacy and safety of four ablation procedures including RFA, CBA, RMN and PFA.

PFA performed best in preventing recurrence of AF and other AT, followed by RMN, CBA, and RFA. PFA is an emerging ablation technique that selectively ablates myocardial tissue by applying a high-voltage pulsed electric field to cardiomyocytes, resulting in electroporation of the cell membrane. The advantage of PFA is that it is tissue-selective, effectively ablating cardiomyocytes without damaging surrounding tissues, which may be associated with its low recurrence rate (36). RMN is suboptimal in preventing recurrence of AF. The precise control of the catheter through the magnetic navigation system enables RMN to minimize human error, thus improving the accuracy and success rate of ablation (37). However, the operational complexity and long learning curve of RMN may limit its wide application. Our study showed that CBA was slightly more effective than RFA, which may be related to the balloon design of CBA being able to better adhere to the pulmonary vein orifices (38). However, the long-term efficacy and safety of RFA, the most established ablation technique, has been widely validated. The excellent performance of PFA in preventing recurrence of AF makes it an important option for future AF ablation. However, the long-term efficacy of PFA needs to be further validated.

In terms of complication rate, RMN had the lowest complication rate while CBA had the highest complication rate. The low complication rate of RMN may be attributed to its precise navigation system and stable catheterization. The magnetic navigation system reduces the risk of perforation and pericardial tamponade by minimizing mechanical contact between the catheter and the inner wall of the cardiac chambers. In addition, the automated operation of the RMN reduces operator error and further improves interventional safety. PFA has a lower complication rate, which may be related to its tissue selectivity. PFA causes less damage to the surrounding tissues, thus reducing the risk of complications (39). However, the high voltage pulses of PFA may trigger muscle contractions and pain and need to be performed under anesthesia or sedation. The advantages of RMN and PFA in reducing complication rates make them the preferred procedures for high-risk AF patients.

This network meta-analysis, PFA had the shortest operative time, whereas RMN had the longest operative time. PFA is capable of completing an ablation point in a matter of seconds, thereby significantly reducing the time to pulmonary vein isolation. The rapid ablation characteristics of PFA allow for a significantly shorter procedure time than other procedures. The longer operative time for RMN may be related to its complex procedure and long learning curve. The setup and calibration of the magnetic navigation system requires additional time and specialized training for the operator to become proficient (40). The procedure times for CBA and RFA are intermediate between those for PFA and RMN. The balloon design of CBA allows for rapid completion of pulmonary vein isolation, but its longer cryo-cycle may increase the procedure time (4). The procedure time for RFA depends on the experience of the operator and the ablation strategy. The short procedure time of PFA makes it a representative of high-efficiency ablation, especially for patients with limited procedure time. However, the longer procedure time of RMN may limit its use in busy clinical settings.

The results of this network meta-analysis showed that RMN had the shortest fluoroscopy time, while PFA had the longest perspective time. Magnetic navigation systems reduce the reliance on x-ray fluoroscopy, thereby significantly reducing radiation exposure. This has important implications for both the operator and the patient, especially during prolonged procedures. PFA requires precise positioning of the ablation catheter and therefore longer fluoroscopy times. This may increase the risk of radiation exposure to both the operator and the patient, especially in complex cases. The low radiation exposure properties of RMN make it a preferred procedure for radiation-sensitive patients, such as pregnant women and young patients. However, the high fluoroscopic demands of PFA need to be optimized by technical improvements.

For the future direction, firstly, conduct more long-term follow-up studies to validate the long-term efficacy of PFA; secondly, optimize the RMN technique to shorten the procedure time and reduce the learning curve; and finally, explore individualized ablation strategies to develop an optimal treatment plan by combining patient characteristics and operator experience.

Our network meta-analysis has several limitations. First, the effect sizes of some interventions, especially those based on small sample sizes or with significant heterogeneity, should be interpreted with caution. The confidence intervals for these effect sizes are often wide, indicating a certain degree of uncertainty in the estimated effects. This variability suggests that the true effects may differ substantially, and future studies require larger and more homogeneous samples to confirm these findings. Second, some of the studies included in this network meta-analysis have poor methodological quality, particularly the lack of a double-blind design, which may introduce bias. This is because blinding of participants and assessors is inherently challenging. Such limitations may introduce potential bias and weaken the strength of the evidence presented. Third, the present study included both RCT and observational studies, which may introduce potential selection bias, confounding bias, and methodological heterogeneity, thereby affecting the stability of the pooled results to a certain extent. Meanwhile, there was substantial heterogeneity in baseline characteristics and clinical profiles of patients across different studies. The follow-up duration was relatively short in most included studies, which limited the comprehensive assessment of long-term efficacy and safety. In addition, the definition and reporting standards of perioperative complications varied considerably across studies, which may result in the underreporting of complications or inconsistent evaluation of clinical outcomes. Furthermore, the number of high-quality RCT and PSM studies remains limited, especially for emerging technologies such as RMN and PFA. The relatively insufficient quantity and variety of direct comparative evidence restrict the stability and reliability of the network estimates. Fourth, owing to the above constraints, the conclusions of this study are still preliminary. Future large-sample, multicenter, head-to-head RCT with long follow-up durations are urgently needed to validate the long-term efficacy, safety, and comparative effectiveness of different ablation strategies, so as to further improve the validity and clinical generalizability of the evidence.

## 5 Conclusion

Our study demonstrated that PFA excelled in preventing AF recurrence and reducing procedure duration, but had a longer fluoroscopy duration; RMN was advantageous in reducing complications and minimizing fluoroscopy duration, but had a longer procedure duration; and CBA and RFA were moderate in each of the metrics. Therefore, the choice of ablation technique needs to be weighed against the specific clinical needs and the patient's situation. If success rate and procedure time are prioritized, PFA may be the best choice; if complication rate and fluoroscopy duration are more important, RMN is more appropriate.

## Data Availability

The original contributions presented in the study are included in the article/Supplementary Material, further inquiries can be directed to the corresponding author.
