# Supplementary material for: Comparative efficacy and safety of catheter ablation technologies for atrial fibrillation: a systematic review and network meta-analysis
Source: Front Cardiovasc Med. 2026 Apr 14;13:1667194. doi: 10.3389/fcvm.2026.1667194 (PMC13120965; doi:10.3389/fcvm.2026.1667194)
Supplement: Supplementary file 1 [file Datasheet1.docx]

Supplementary Material

**Appendix 1**

**Comparative efficacy and safety of catheter ablation technologies for atrial fibrillation: A systematic review and network meta-analysis**

Ruiting Feng^1,2^, Jia Gao^1^, Yajie Guo^1,2^, Yuli Guo^1,2^, Rui Wang^1^, Min Guo^1^

^1^Department of Cardiology, First Hospital of Shanxi Medical University, Taiyuan, Shanxi, China

^2^The First Clinical Medical College, Shanxi Medical University, Taiyuan, Shanxi, China

Correspondence: Min Guo ([guomin162@163.com](mailto:guomin162@163.com))

Corresponding author

Min Guo

Email: [guomin162@163.com](mailto:guomin162@163.com)

S1. Search strategy

Search: (("Atrial Fibrillation"[Mesh]) OR (((((((((((((((((((((((((Atrial Fibrillations[Title/Abstract]) OR (Fibrillation, Atrial[Title/Abstract])) OR (Fibrillations, Atrial[Title/Abstract])) OR (Auricular Fibrillation[Title/Abstract])) OR (Auricular Fibrillations[Title/Abstract])) OR (Fibrillation, Auricular[Title/Abstract])) OR (Fibrillations,Auricular[Title/Abstract])) OR (Persistent Atrial Fibrillation[Title/Abstract])) OR (Atrial Fibrillation, Persistent[Title/Abstract])) OR (Atrial Fibrillations, Persistent[Title/Abstract])) OR (Fibrillation, Persistent Atrial[Title/Abstract])) OR (Fibrillations, Persistent Atrial[Title/Abstract])) OR (Persistent Atrial Fibrillations[Title/Abstract])) OR (Familial Atrial Fibrillation[Title/Abstract])) OR (Atrial Fibrillation, Familial[Title/Abstract])) OR (Atrial Fibrillations, Familial[Title/Abstract])) OR (Familial Atrial Fibrillations[Title/Abstract])) OR (Fibrillation, Familial Atrial[Title/Abstract])) OR (Fibrillations, Familial Atrial[Title/Abstract])) OR (Paroxysmal Atrial Fibrillation[Title/Abstract])) OR (Atrial Fibrillation,Paroxysmal[Title/Abstract])) OR (Atrial Fibrillations, Paroxysmal[Title/Abstract])) OR (Fibrillation, Paroxysmal Atrial[Title/Abstract])) OR (Fibrillations, Paroxysmal Atrial[Title/Abstract])) OR (Paroxysmal Atrial Fibrillations[Title/Abstract]))) AND (((((((((((("Catheter Ablation"[Mesh]) OR ((((((((((((((((((((((Ablation, Catheter[Title/Abstract]) OR (Ablation, Transvenous Electric[Title/Abstract])) OR (Electric Ablation,Transvenous[Title/Abstract])) OR (Transvenous Electric Ablation[Title/Abstract])) OR (Ablation, Transvenous Electrical[Title/Abstract])) OR (Electrical Ablation, Transvenous[Title/Abstract])) OR (Transvenous Electrical Ablation[Title/Abstract])) OR (Catheter Ablation, Electric[Title/Abstract])) OR (Electric Catheter Ablation[Title/Abstract])) OR (Ablation, Electric Catheter[Title/Abstract])) OR (Catheter Ablation, Electrical[Title/Abstract])) OR (Ablation, Electrical Catheter[Title/Abstract])) OR (Electrical Catheter Ablation[Title/Abstract])) OR (Catheter Ablation, Percutaneous[Title/Abstract])) OR (Percutaneous Catheter Ablation[Title/Abstract])) OR (Ablation, Percutaneous Catheter[Title/Abstract])) OR (Catheter Ablation, Radiofrequency[Title/Abstract])) OR (Radiofrequency Catheter Ablation[Title/Abstract])) OR (Ablation, Radiofrequency Catheter[Title/Abstract])) OR (Catheter Ablation, Transvenous[Title/Abstract])) OR (Transvenous Catheter Ablation[Title/Abstract])) OR (Ablation, Transvenous Catheter[Title/Abstract]))) AND (((((remote magnetic[Title/Abstract]) OR (remote magnetic catheter navigation[Title/Abstract])) OR (remote magnetic navigation[Title/Abstract])) OR (magnetic navigation system[Title/Abstract])) OR (robotic magnetic navigation[Title/Abstract]))) OR ((("Catheter Ablation"[Mesh]) OR ((((((((((((((((((((((Ablation, Catheter[Title/Abstract]) OR (Ablation, Transvenous Electric[Title/Abstract])) OR (Electric Ablation, Transvenous[Title/Abstract])) OR (Transvenous Electric Ablation[Title/Abstract])) OR (Ablation, Transvenous Electrical[Title/Abstract])) OR (Electrical Ablation, Transvenous[Title/Abstract])) OR (Transvenous Electrical Ablation[Title/Abstract])) OR (Catheter Ablation, Electric[Title/Abstract])) OR (Electric CatheterAblation[Title/Abstract])) OR (Ablation, Electric Catheter[Title/Abstract])) OR (Catheter Ablation,Electrical[Title/Abstract])) OR (Ablation, Electrical Catheter[Title/Abstract])) OR (Electrical Catheter Ablation[Title/Abstract])) OR (Catheter Ablation, Percutaneous[Title/Abstract])) OR (Percutaneous Catheter Ablation[Title/Abstract])) OR (Ablation, Percutaneous Catheter[Title/Abstract])) OR (Catheter Ablation, Radiofrequency[Title/Abstract])) OR (Radiofrequency Catheter Ablation[Title/Abstract])) OR (Ablation, Radiofrequency Catheter[Title/Abstract])) OR (Catheter Ablation, Transvenous[Title/Abstract])) OR (Transvenous Catheter Ablation[Title/Abstract])) OR (Ablation, Transvenous Catheter[Title/Abstract]))) AND (((Cryoballoon[Title/Abstract]) OR (cryoballoon ablation[Title/Abstract])) OR (Cryoballoon Catheter Ablation[Title/Abstract])))) OR ((("Catheter Ablation"[Mesh]) OR ((((((((((((((((((((((Ablation, Catheter[Title/Abstract]) OR (Ablation, Transvenous Electric[Title/Abstract])) OR (Electric Ablation, Transvenous[Title/Abstract])) OR (Transvenous Electric Ablation[Title/Abstract])) OR (Ablation, Transvenous Electrical[Title/Abstract])) OR (Electrical Ablation, Transvenous[Title/Abstract])) OR (Transvenous Electrical Ablation[Title/Abstract])) OR (Catheter Ablation, Electric[Title/Abstract])) OR (Electric Catheter Ablation[Title/Abstract])) OR (Ablation, Electric Catheter[Title/Abstract])) OR (Catheter Ablation, Electrical[Title/Abstract])) OR (Ablation, Electrical Catheter[Title/Abstract])) OR (Electrical Catheter Ablation[Title/Abstract])) OR (Catheter Ablation, Percutaneous[Title/Abstract])) OR (Percutaneous Catheter Ablation[Title/Abstract])) OR (Ablation, Percutaneous Catheter[Title/Abstract])) OR (Catheter Ablation,Radiofrequency[Title/Abstract])) OR (Radiofrequency Catheter Ablation[Title/Abstract])) OR (Ablation, Radiofrequency Catheter[Title/Abstract])) OR (Catheter Ablation, Transvenous[Title/Abstract])) OR (Transvenous Catheter Ablation[Title/Abstract])) OR (Ablation, Transvenous Catheter[Title/Abstract]))) AND (((Pulsed field ablation[Title/Abstract]) OR (Pulsed Radiofrequency Ablation[Title/Abstract])) OR (Pulsed electric field ablation[Title/Abstract])))) OR ((((((remote magnetic[Title/Abstract]) OR (remote magnetic catheter navigation[Title/Abstract])) OR (remote magnetic navigation[Title/Abstract])) OR (magnetic navigation system[Title/Abstract])) OR (robotic magnetic navigation[Title/Abstract])) AND (((Cryoballoon[Title/Abstract]) OR (cryoballoon ablation[Title/Abstract])) OR (Cryoballoon Catheter Ablation[Title/Abstract])))) OR (("remote magnetic"[Title/Abstract] OR "remote magnetic catheter navigation"[Title/Abstract] OR "remote magnetic navigation"[Title/Abstract] OR "magnetic navigation system"[Title/Abstract] OR "robotic magnetic navigation"[Title/Abstract]) AND ("pulsed field ablation"[Title/Abstract] OR "pulsed radiofrequency ablation"[Title/Abstract] OR "pulsed electric field ablation"[Title/Abstract]))) OR ((((Cryoballoon[Title/Abstract]) OR (cryoballoon ablation[Title/Abstract])) OR (Cryoballoon Catheter Ablation[Title/Abstract])) AND (((Pulsed field ablation[Title/Abstract]) OR (Pulsed Radiofrequency Ablation[Title/Abstract])) OR (Pulsed electric field ablation[Title/Abstract])))) OR (((("Catheter Ablation"[Mesh]) OR ((((((((((((((((((((((Ablation,Catheter[Title/Abstract]) OR (Ablation, Transvenous Electric[Title/Abstract])) OR (Electric Ablation,Transvenous[Title/Abstract])) OR (Transvenous Electric Ablation[Title/Abstract])) OR (Ablation,Transvenous Electrical[Title/Abstract])) OR (Electrical Ablation, Transvenous[Title/Abstract])) OR (Transvenous Electrical Ablation[Title/Abstract])) OR (Catheter Ablation, Electric[Title/Abstract])) OR (Electric Catheter Ablation[Title/Abstract])) OR (Ablation, Electric Catheter[Title/Abstract])) OR (Catheter Ablation, Electrical[Title/Abstract])) OR (Ablation, Electrical Catheter[Title/Abstract])) OR (Electrical Catheter Ablation[Title/Abstract])) OR (Catheter Ablation, Percutaneous[Title/Abstract])) OR (Percutaneous Catheter Ablation[Title/Abstract])) OR (Ablation, Percutaneous Catheter[Title/Abstract])) OR (Catheter Ablation, Radiofrequency[Title/Abstract])) OR (Radiofrequency Catheter Ablation[Title/Abstract])) OR (Ablation, Radiofrequency Catheter[Title/Abstract])) OR (Catheter Ablation, Transvenous[Title/Abstract])) OR (Transvenous Catheter Ablation[Title/Abstract])) OR (Ablation, Transvenous Catheter[Title/Abstract]))) AND (((((remote magnetic[Title/Abstract]) OR (remote magnetic catheter navigation[Title/Abstract])) OR (remote magnetic navigation[Title/Abstract])) OR (magnetic navigation system[Title/Abstract])) OR (robotic magnetic navigation[Title/Abstract]))) AND (((Cryoballoon[Title/Abstract]) OR (cryoballoon ablation[Title/Abstract])) OR (Cryoballoon Catheter Ablation[Title/Abstract])))) OR (("Catheter Ablation"[MeSH Terms] OR ("ablation catheter"[Title/Abstract] OR (("ablate"[All Fields] OR "ablated"[All Fields] OR "ablates"[All Fields] OR "ablating"[All Fields] OR "Ablation"[All Fields] OR "ablational"[All Fields] OR "ablations"[All Fields]) AND "transvenous electric"[Title/Abstract]) OR (("electricity"[MeSH Terms] OR "electricity"[All Fields] OR "Electric"[All Fields] OR "Electrical"[All Fields] OR "electrically"[All Fields] OR "electrics"[All Fields]) AND "ablation transvenous"[Title/Abstract]) OR "transvenous electric ablation"[Title/Abstract] OR (("ablate"[All Fields] OR "ablated"[All Fields] OR "ablates"[All Fields] OR "ablating"[All Fields] OR "Ablation"[All Fields] OR "ablational"[All Fields] OR "ablations"[All Fields]) AND "transvenous electrical"[Title/Abstract]) OR (("electricity"[MeSH Terms] OR "electricity"[All Fields] OR "Electric"[All Fields] OR "Electrical"[All Fields] OR "electrically"[All Fields] OR "electrics"[All Fields]) AND "ablation transvenous"[Title/Abstract]) OR "transvenous electrical ablation"[Title/Abstract] OR (("catheter s"[All Fields] OR "catheters"[MeSH Terms] OR "catheters"[All Fields] OR "Catheter"[All Fields]) AND "ablation electric"[Title/Abstract]) OR "electric catheter ablation"[Title/Abstract] OR (("ablate"[All Fields] OR "ablated"[All Fields] OR "ablates"[All Fields] OR "ablating"[All Fields] OR "Ablation"[All Fields] OR "ablational"[All Fields] OR "ablations"[All Fields]) AND "electric catheter"[Title/Abstract]) OR "catheter ablation electrical"[Title/Abstract] OR (("ablate"[All Fields] OR "ablated"[All Fields] OR "ablates"[All Fields] OR "ablating"[All Fields] OR "Ablation"[All Fields] OR "ablational"[All Fields] OR "ablations"[All Fields]) AND "electrical catheter"[Title/Abstract]) OR "electrical catheter ablation"[Title/Abstract] OR "catheter ablation percutaneous"[Title/Abstract] OR "percutaneous catheter ablation"[Title/Abstract] OR (("ablate"[All Fields] OR "ablated"[All Fields] OR "ablates"[All Fields] OR "ablating"[All Fields] ORAblation"[All Fields] OR "ablational"[All Fields] OR "ablations"[All Fields]) AND "percutaneous catheter"[Title/Abstract]) OR "catheter ablation radiofrequency"[Title/Abstract] OR "radiofrequency catheter ablation"[Title/Abstract] OR "ablation radiofrequency catheter"[Title/Abstract] OR (("catheter s"[All Fields] OR "catheters"[MeSH Terms] OR "catheters"[All Fields] OR "Catheter"[All Fields]) AND "ablation transvenous"[Title/Abstract]) OR "transvenous catheter ablation"[Title/Abstract] OR (("ablate"[All Fields] OR "ablated"[All Fields] OR "ablates"[All Fields] OR "ablating"[All Fields] OR "Ablation"[All Fields] OR "ablational"[All Fields] OR "ablations"[All Fields]) AND "transvenous catheter"[Title/Abstract]))) AND ("remote magnetic"[Title/Abstract] OR "remote magnetic catheter navigation"[Title/Abstract] OR "remote magnetic navigation"[Title/Abstract] OR "magnetic navigation system"[Title/Abstract] OR "robotic magnetic navigation"[Title/Abstract]) AND ("pulsed field ablation"[Title/Abstract] OR "pulsed radiofrequency ablation"[Title/Abstract] OR "pulsed electric field ablation"[Title/Abstract]))) OR (("remote magnetic"[Title/Abstract] OR "remote magnetic catheter navigation"[Title/Abstract] OR "remote magnetic navigation"[Title/Abstract] OR "magnetic navigation system"[Title/Abstract] OR "robotic magnetic navigation"[Title/Abstract]) AND ("Cryoballoon"[Title/Abstract] OR "cryoballoon ablation"[Title/Abstract] OR "cryoballoon catheter ablation"[Title/Abstract]) AND ("pulsed field ablation"[Title/Abstract] OR "pulsed radiofrequency ablation"[Title/Abstract] OR "pulsed electric field ablation"[Title/Abstract]))) OR (("Catheter Ablation"[MeSH Terms] OR ("ablation catheter"[Title/Abstract] OR (("ablate"[All Fields] OR "ablated"[All Fields] OR "ablates"[All Fields] OR "ablating"[All Fields] OR "Ablation"[All Fields] OR "ablational"[All Fields] OR "ablations"[All Fields]) AND "transvenous electric"[Title/Abstract]) OR (("electricity"[MeSH Terms] OR "electricity"[All Fields] OR "Electric"[All Fields] OR "Electrical"[All Fields] OR "electrically"[All Fields] OR "electrics"[All Fields]) AND "ablation transvenous"[Title/Abstract]) OR "transvenous electric ablation"[Title/Abstract] OR (("ablate"[All Fields] OR "ablated"[All Fields] OR "ablates"[All Fields] OR "ablating"[All Fields] OR "Ablation"[All Fields] OR "ablational"[All Fields] OR "ablations"[All Fields]) AND "transvenous electrical"[Title/Abstract]) OR (("electricity"[MeSH Terms] OR "electricity"[All Fields] OR "Electric"[All Fields] OR "Electrical"[All Fields] OR "electrically"[All Fields] OR "electrics"[All Fields]) AND "ablation transvenous"[Title/Abstract]) OR "transvenous electrical ablation"[Title/Abstract] OR (("catheter s"[All Fields] OR "catheters"[MeSH Terms] OR "catheters"[All Fields] OR "Catheter"[All Fields]) AND "ablation electric"[Title/Abstract]) OR "electric catheter ablation"[Title/Abstract] OR (("ablate"[All Fields] OR "ablated"[All Fields] OR "ablates"[All Fields] OR "ablating"[All Fields] OR "Ablation"[All Fields] OR "ablational"[All Fields] OR "ablations"[All Fields]) AND "electric catheter"[Title/Abstract]) OR "catheter ablation electrical"[Title/Abstract] OR (("ablate"[All Fields] OR "ablated"[All Fields] OR "ablates"[All Fields] OR "ablating"[All Fields] OR "Ablation"[All Fields] OR "ablational"[All Fields] OR "ablations"[All Fields]) AND "electrical catheter"[Title/Abstract]) OR "electrical catheter ablation"[Title/Abstract] OR "catheter ablation percutaneous"[Title/Abstract] OR "percutaneous catheter ablation"[Title/Abstract] OR (("ablate"[All Fields] OR "ablated"[All Fields] OR "ablates"[All Fields] OR "ablating"[All Fields] OR "Ablation"[All Fields] OR "ablational"[All Fields] OR "ablations"[All Fields]) AND "percutaneous catheter"[Title/Abstract]) OR "catheter ablation radiofrequency"[Title/Abstract] OR "radiofrequency catheter ablation"[Title/Abstract] OR "ablation radiofrequency catheter"[Title/Abstract] OR (("catheter s"[All Fields] OR "catheters"[MeSH Terms] OR "catheters"[All Fields] OR "Catheter"[All Fields]) AND "ablation transvenous"[Title/Abstract]) OR "transvenous catheter ablation"[Title/Abstract] OR (("ablate"[All Fields] OR "ablated"[All Fields] OR "ablates"[All Fields] OR "ablating"[All Fields] OR "Ablation"[All Fields] OR "ablational"[All Fields] OR "ablations"[All Fields]) AND "transvenous catheter"[Title/Abstract]))) AND ("remote magnetic"[Title/Abstract] OR "remote magnetic catheter navigation"[Title/Abstract] OR "remote magnetic navigation"[Title/Abstract] OR "magnetic navigation system"[Title/Abstract] OR "robotic magnetic navigation"[Title/Abstract]) AND ("Cryoballoon"[Title/Abstract] OR "cryoballoon ablation"[Title/Abstract] OR "cryoballoon catheter ablation"[Title/Abstract]) AND ("pulsed field ablation"[Title/Abstract] OR "pulsed radiofrequency ablation"[Title/Abstract] OR "pulsed electric field ablation"[Title/Abstract])))

## S2. Risk of bias assessment

## Table. S1 The Newcastle-Ottawa Scale (NOS)

| Study | Selection | Comparability | Outcome | Score |
| --- | --- | --- | --- | --- |
| PEDRO PULIDO ADRAG ˜AO 2016 | ⭐⭐⭐⭐ | ⭐ | ⭐⭐⭐ | 8 |
| Domenico G. Della Rocca 2023 | ⭐⭐⭐⭐ | ⭐ | ⭐⭐⭐ | 8 |
| Mario Matta 2018 | ⭐⭐⭐⭐ | ⭐ | ⭐⭐⭐ | 8 |
| Jens Maurhofer 2024 | ⭐⭐⭐⭐ | ⭐ | ⭐⭐⭐ | 8 |

Fig. S1 The Cochrane Collaboration Network's Risk of Bias Assessment Tool


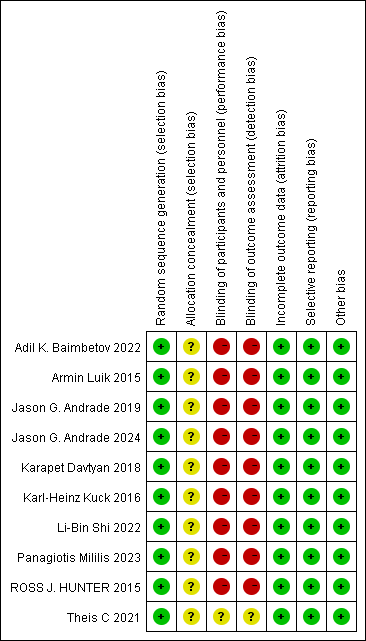





## S3. Outcome-freedom from AF and other AT

Fig. S2 Direct comparison for freedom from AF and other AT


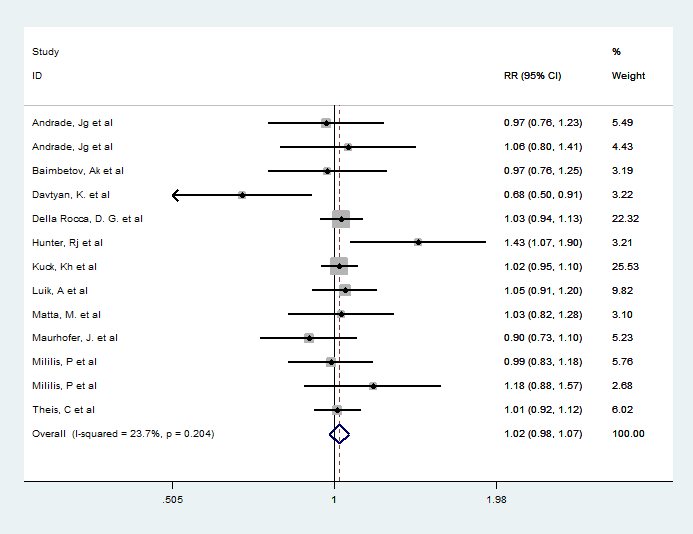


CBA VS RFA


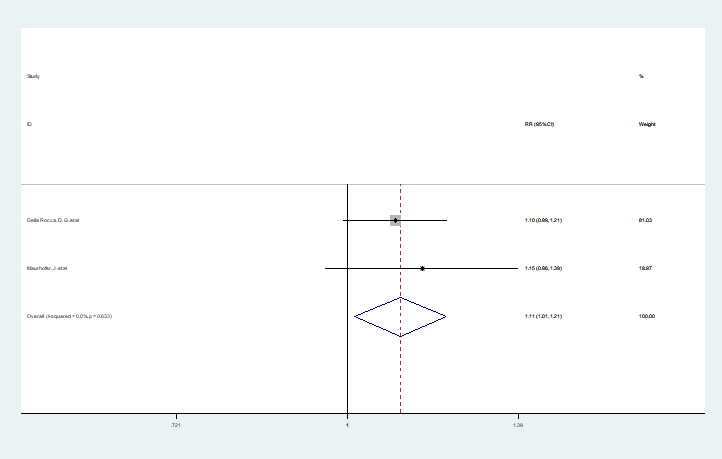


PFA VS RFA


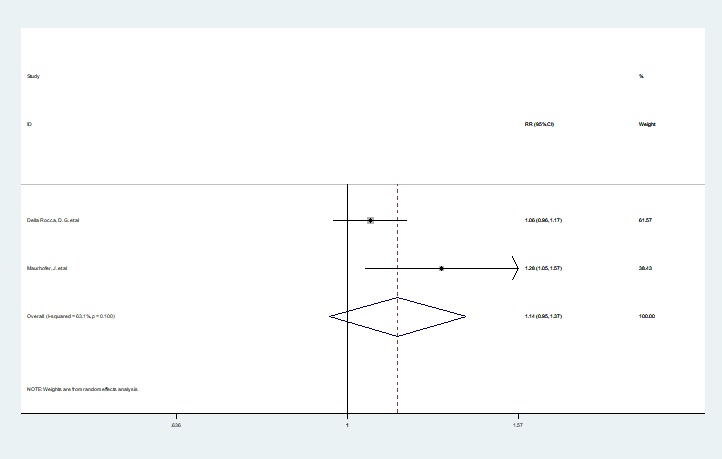


PFA VS CBA

*Note.* RFA means Radiofrequency Ablation; CBA means Cryoballoon Ablation; PFA means Pulsed Field Ablation

Fig. S3 Mesh inconsistency detection of freedom from AF and other AT


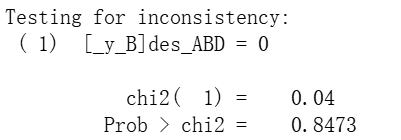


Fig. S4 The ranking of effects for freedom from AF and other AT


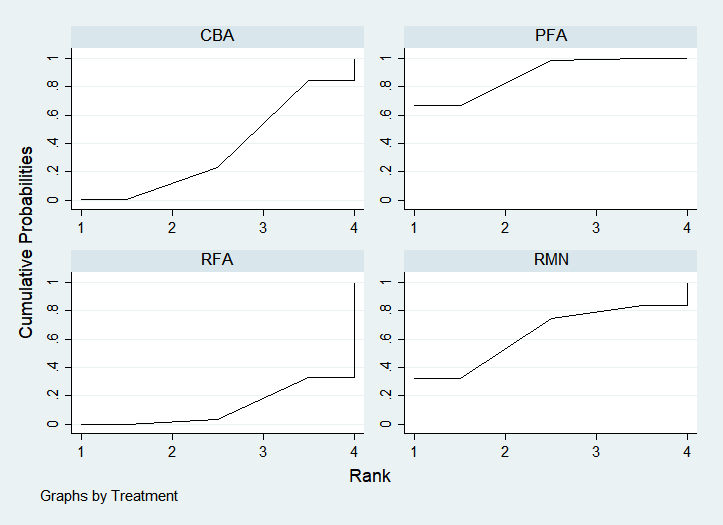


*Note.* RFA means Radiofrequency Ablation; CBA means Cryoballoon Ablation; RMN means Remote Magnetic Navigation Ablation; PFA means Pulsed Field Ablation

Fig. S5 Forest plots of network meta-analysis for freedom from AF and other AT


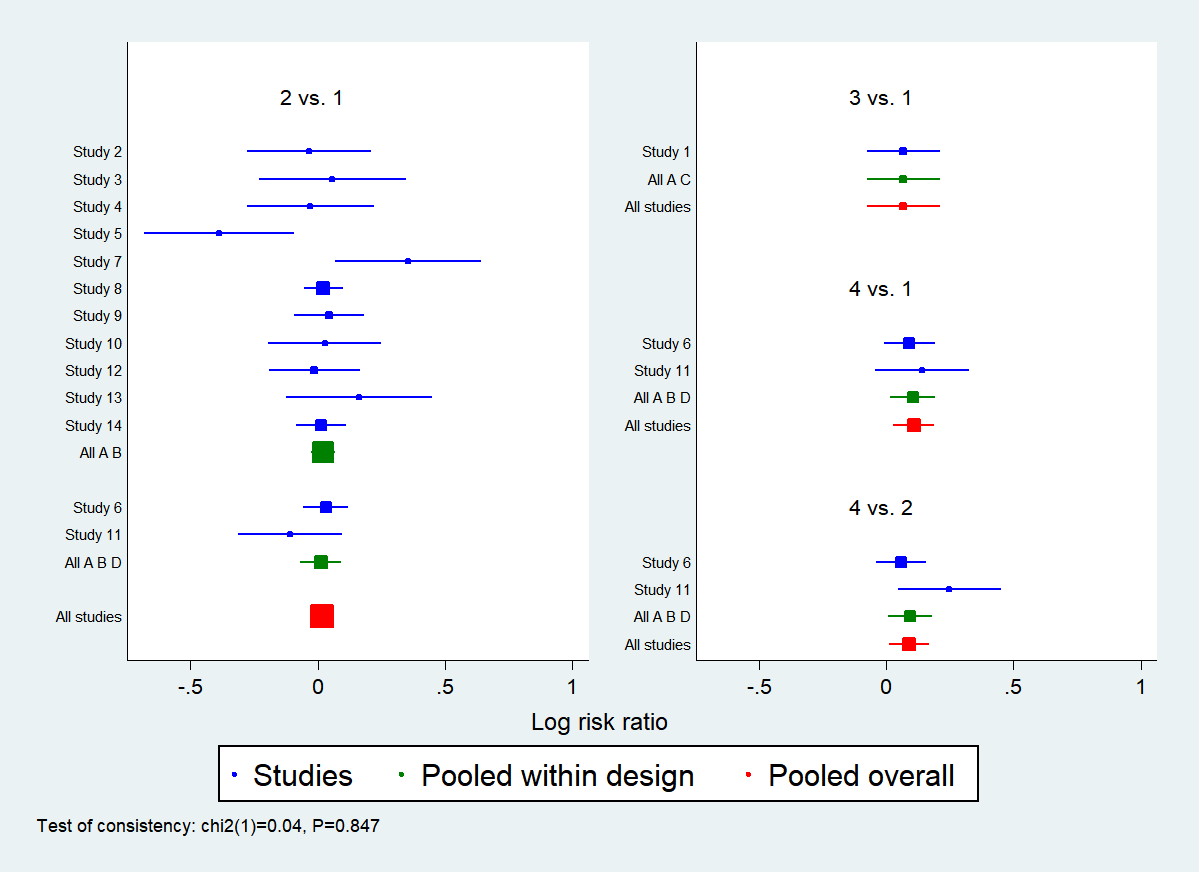


*Note.* 1 means Radiofrequency Ablation (RFA); 2 means Cryoballoon Ablation (CBA); 3 means Remote Magnetic Navigation Ablation (RMN); 4 means Pulised Field Ablation (PFA)

Fig. S6 Funnel plots of freedom from AF and other AT


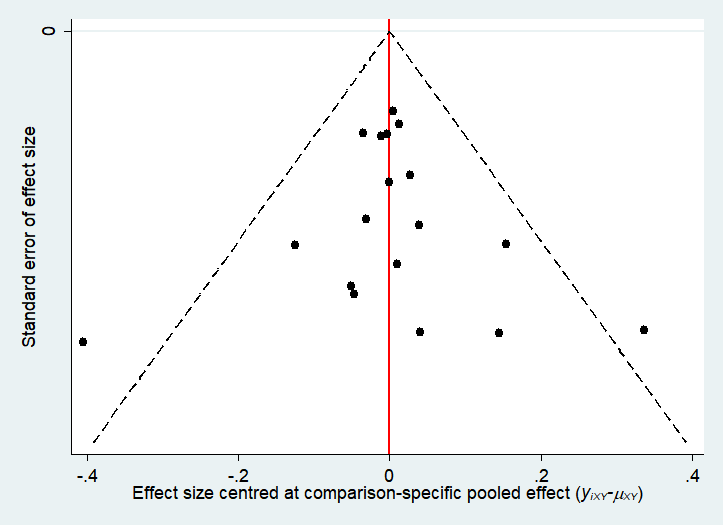


S4. Outcome-complications

Fig. S7 Direct comparison for complications


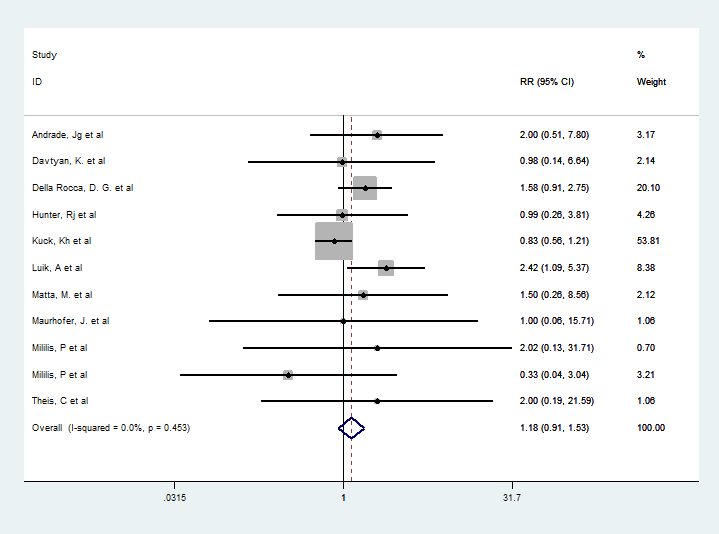


CBA VS RFA


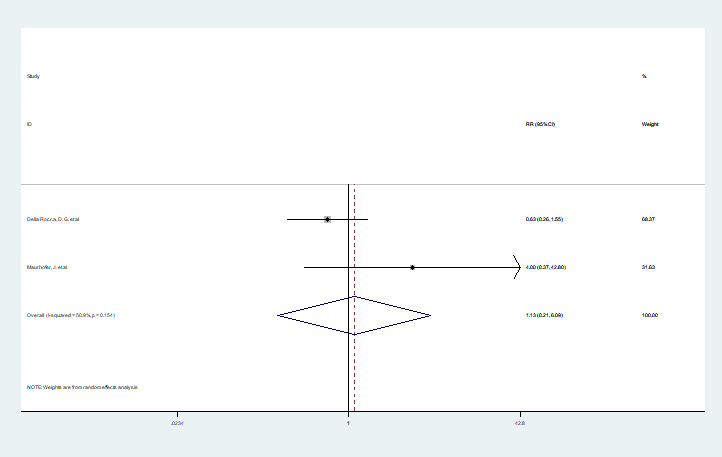


PFA VS RFA


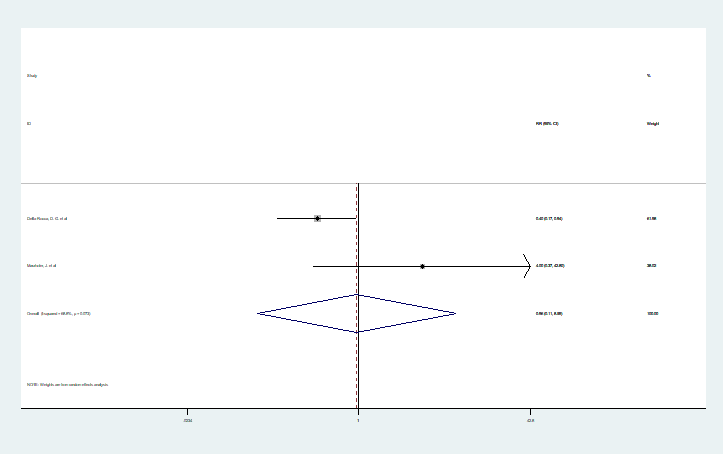


PFA VS CBA

*Note.* RFA means Radiofrequency Ablation; CBA means Cryoballoon Ablation; PFA means Pulsed Field Ablation

Fig. S8 Mesh inconsistency detection of complications


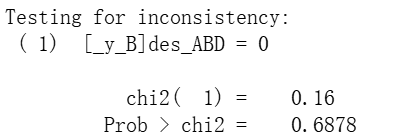


Fig. S9 The ranking of effects for complications


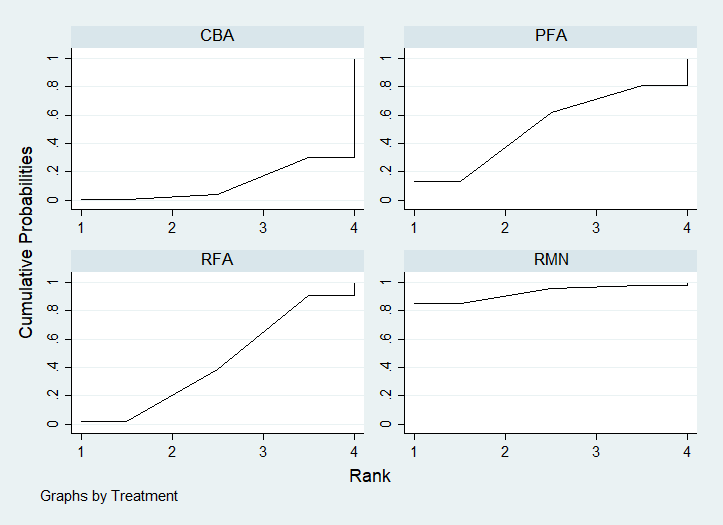


*Note.* RFA means Radiofrequency Ablation; CBA means Cryoballoon Ablation; RMN means Remote Magnetic Navigation Ablation; PFA means Pulsed Field Ablation

Fig. S10 Forest plots of network meta-analysis for complications


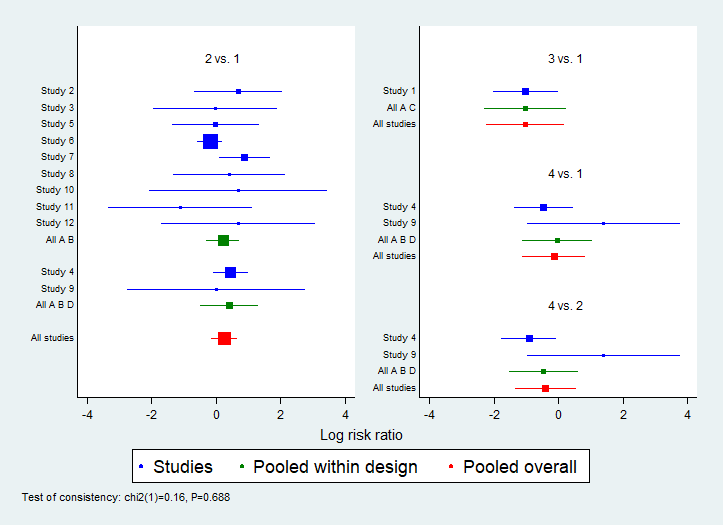


*Note.* 1 means Radiofrequency Ablation (RFA); 2 means Cryoballoon Ablation (CBA); 3 means Remote Magnetic Navigation Ablation (RMN); 4 means Pulised Field Ablation (PFA)

Fig. S11 Funnel plots of complications


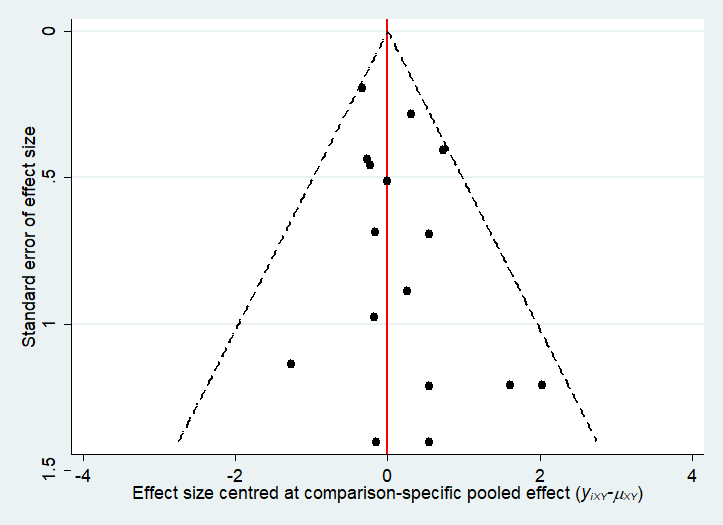


## S5. Outcome-procedure duration

Fig. S12 Direct comparison for procedure duration


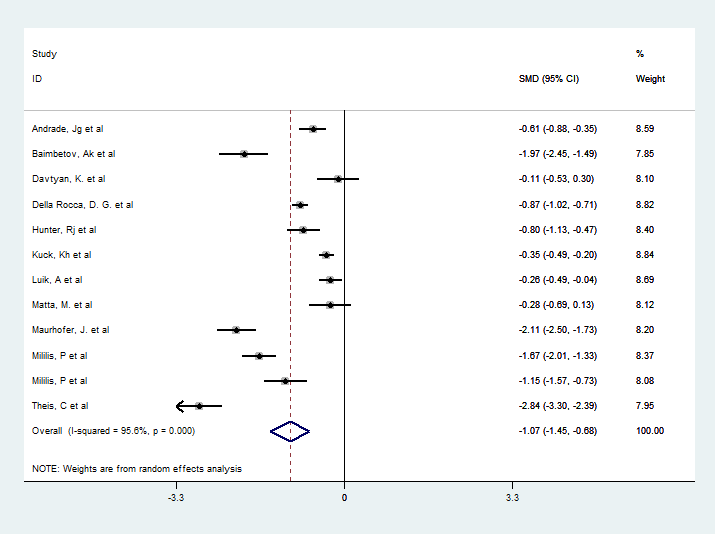


CBA VS RFA


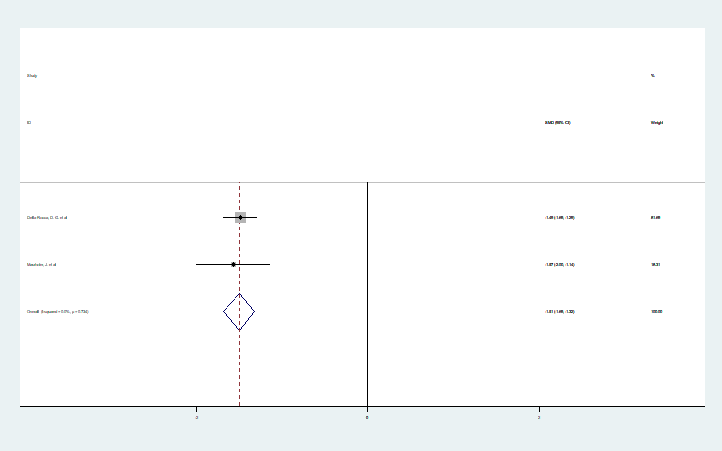


PFA VS RFA


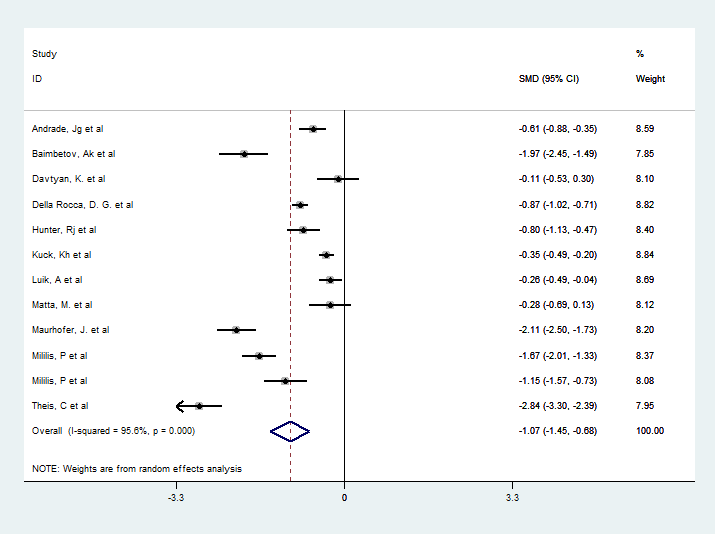


PFA VS CBA

*Note.* RFA means Radiofrequency Ablation; CBA means Cryoballoon Ablation; PFA means Pulsed Field Ablation

Fig. S13 Mesh inconsistency detection of procedure duration


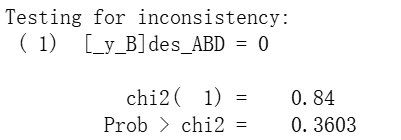


Fig. S14 The ranking of effects for procedure duration


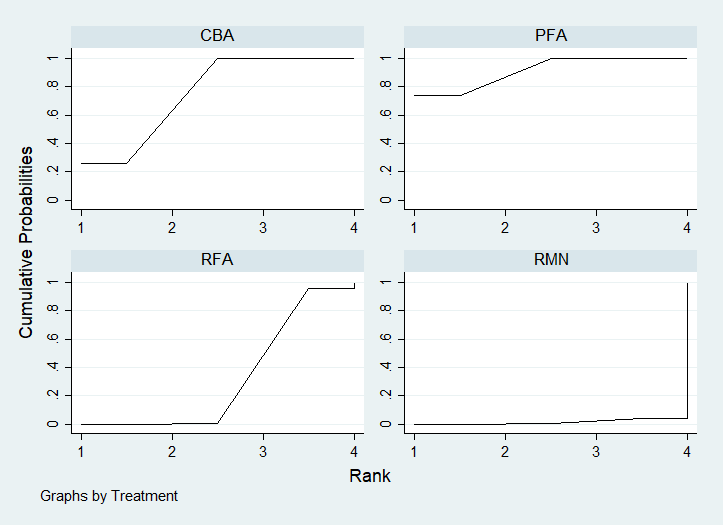


*Note.* RFA means Radiofrequency Ablation; CBA means Cryoballoon Ablation; RMN means Remote Magnetic Navigation Ablation; PFA means Pulsed Field Ablation

Fig. S15 Forest plots of network meta-analysis for procedure duration


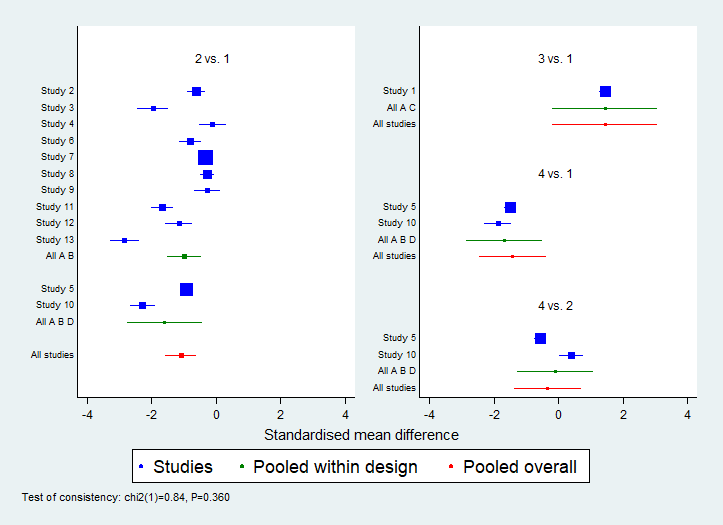


*Note.* 1 means Radiofrequency Ablation (RFA); 2 means Cryoballoon Ablation (CBA); 3 means Remote Magnetic Navigation Ablation (RMN); 4 means Pulised Field Ablation (PFA)

Fig. S16 Funnel plots of procedure duration


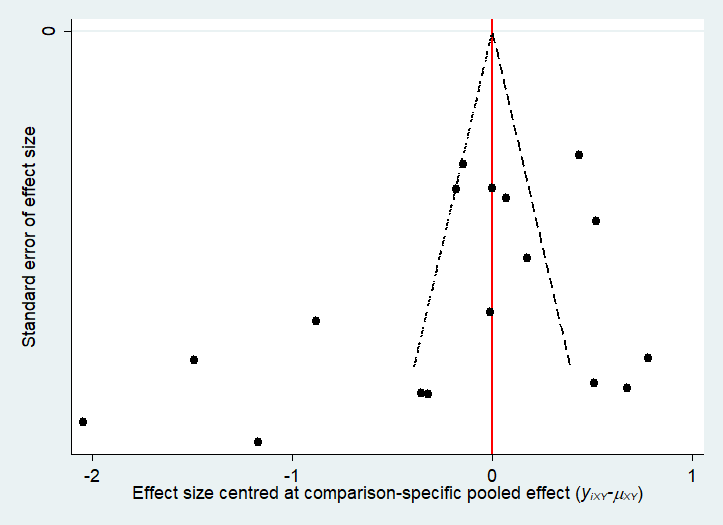


S6. Outcome-fluoroscopy duration

Fig. S17 Direct comparison for fluoroscopy duration


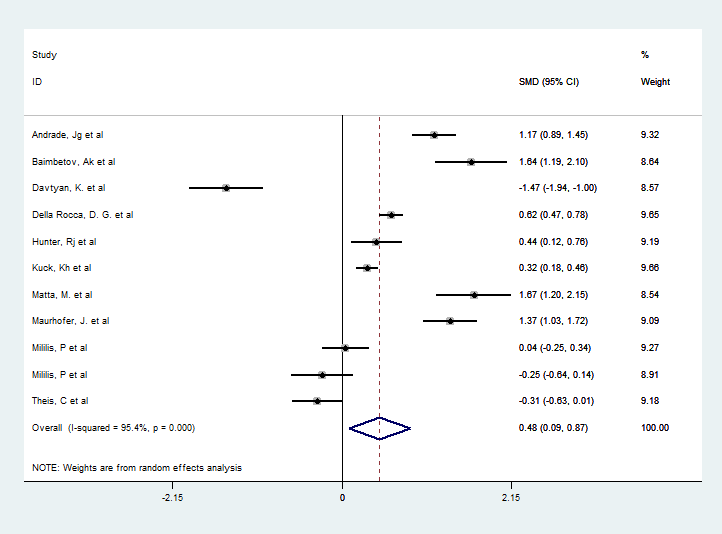


CBA VS RFA


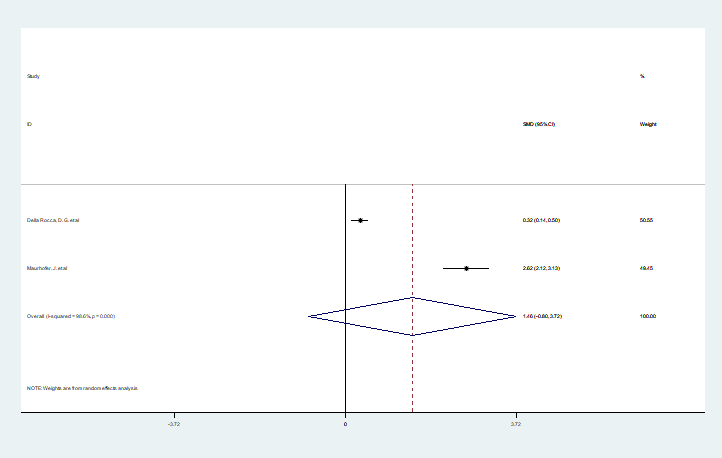


PFA VS RFA


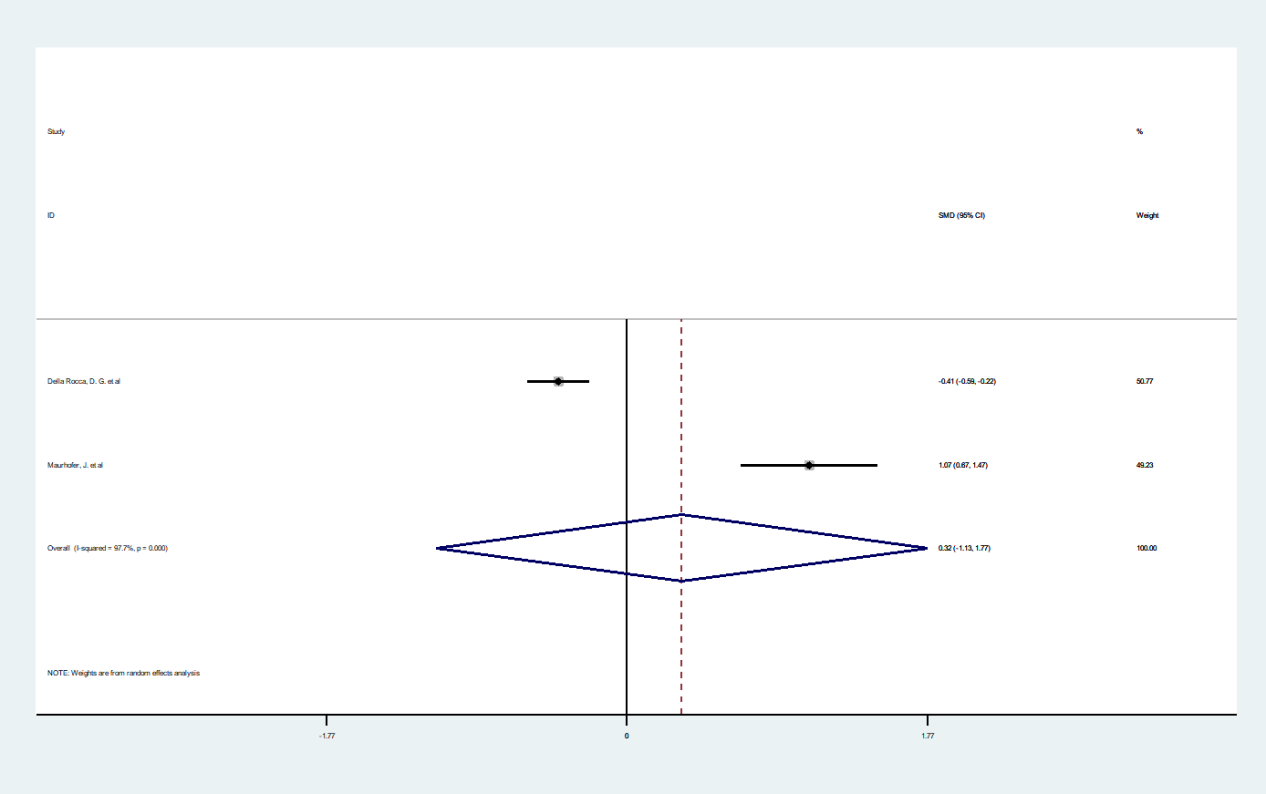


PFA VS CBA

*Note.* RFA means Radiofrequency Ablation; CBA means Cryoballoon Ablation; PFA means Pulsed Field Ablation

Fig. S18 Mesh inconsistency detection of fluoroscopy duration


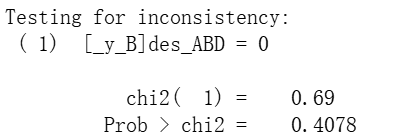


Fig. S19 The ranking of effects for fluoroscopy duration


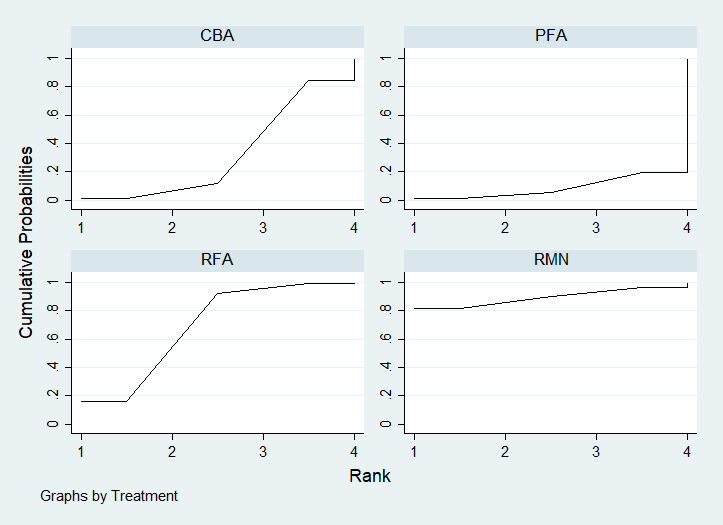


*Note.* RFA means Radiofrequency Ablation; CBA means Cryoballoon Ablation; RMN means Remote Magnetic Navigation Ablation; PFA means Pulsed Field Ablation

Fig. S20 Forest plots of network meta-analysis for fluoroscopy duration


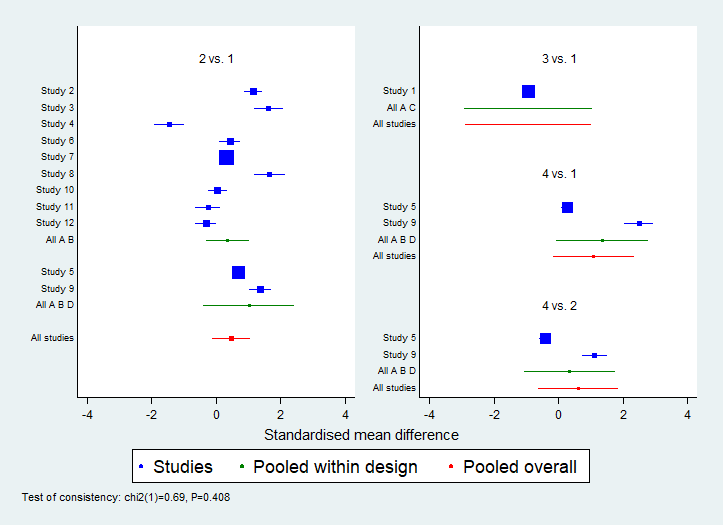


*Note.* 1 means Radiofrequency Ablation (RFA); 2 means Cryoballoon Ablation (CBA); 3 means Remote Magnetic Navigation Ablation (RMN); 4 means Pulised Field Ablation (PFA)

Fig. S21 Funnel plots of fluoroscopy duration


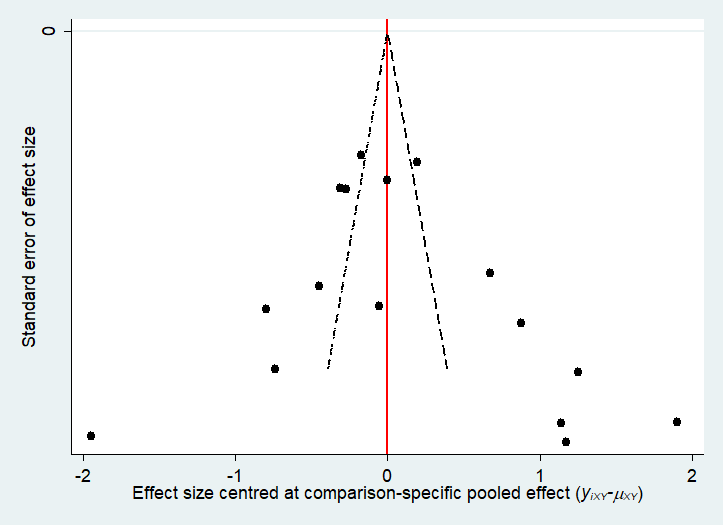


S7. Subgroup analyses

Fig. S22 Subgroup analysis for different type of atrial fibrillation based on the outcome of freedom from AF and other AT.


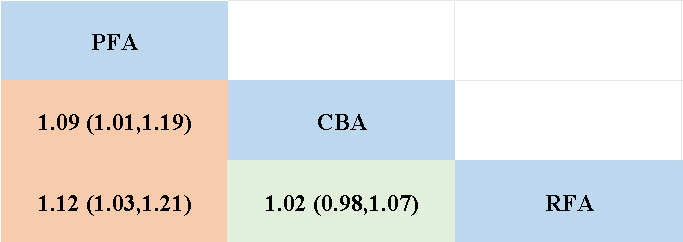


*Note.* Subgroup analysis of paroxysmal atrial fibrillation (PFA)


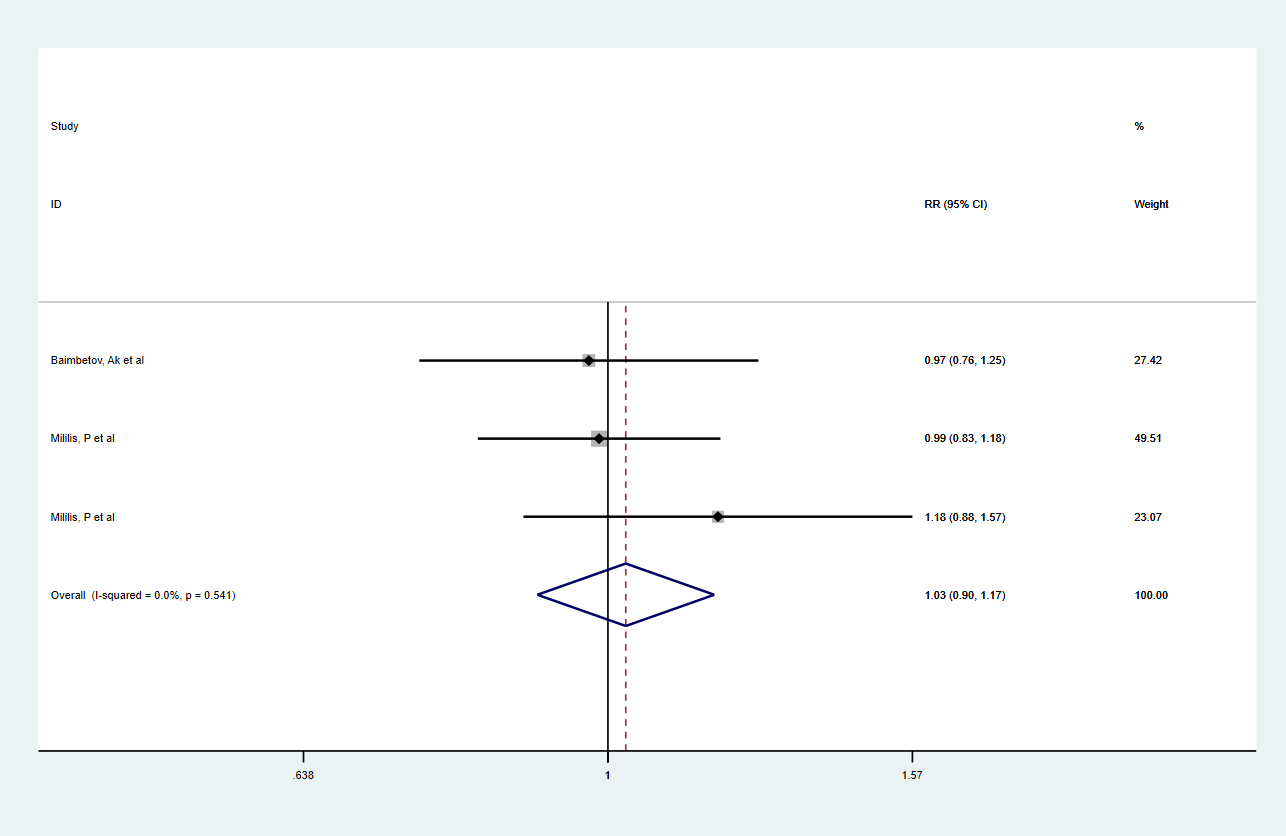


CBA VS RFA

*Note.* Subgroup analysis of persistent atrial fibrillation (PerAF)

RFA means Radiofrequency Ablation; CBA means Cryoballoon Ablation; PFA means Pulsed Field Ablation

Fig. S23 **Inconsistency plot for PAF based on the outcome of freedom from AF and other AT**

**
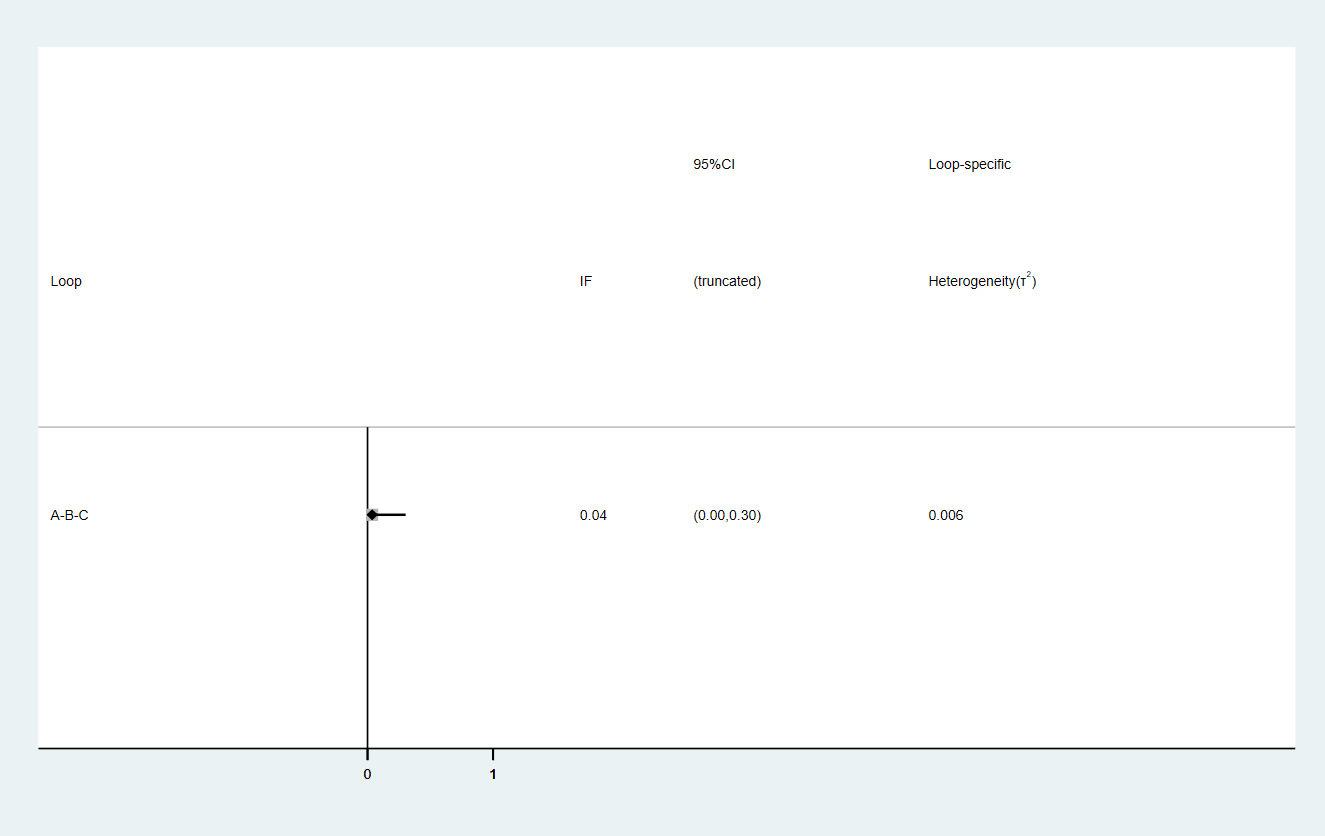
**

*Note. A* means Radiofrequency Ablation (RFA); B means Cryoballoon Ablation (CBA); C means Pulised Field Ablation (PFA)

Fig. S24 Subgroup analysis of different study design based on the outcome of freedom from AF and other AT


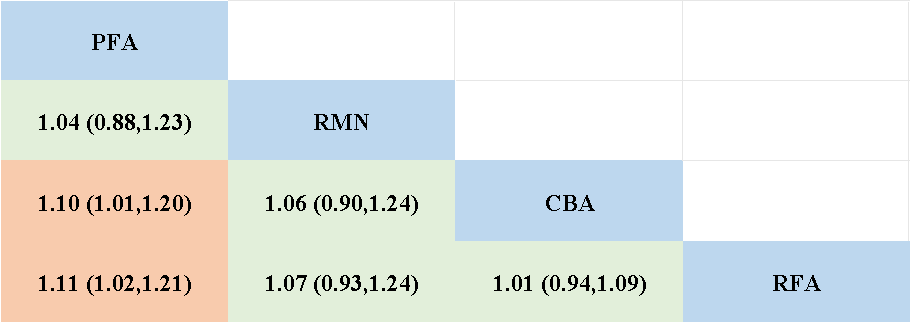


*Note.* Subgroup analysis of propensity-score matched studies (PSM)


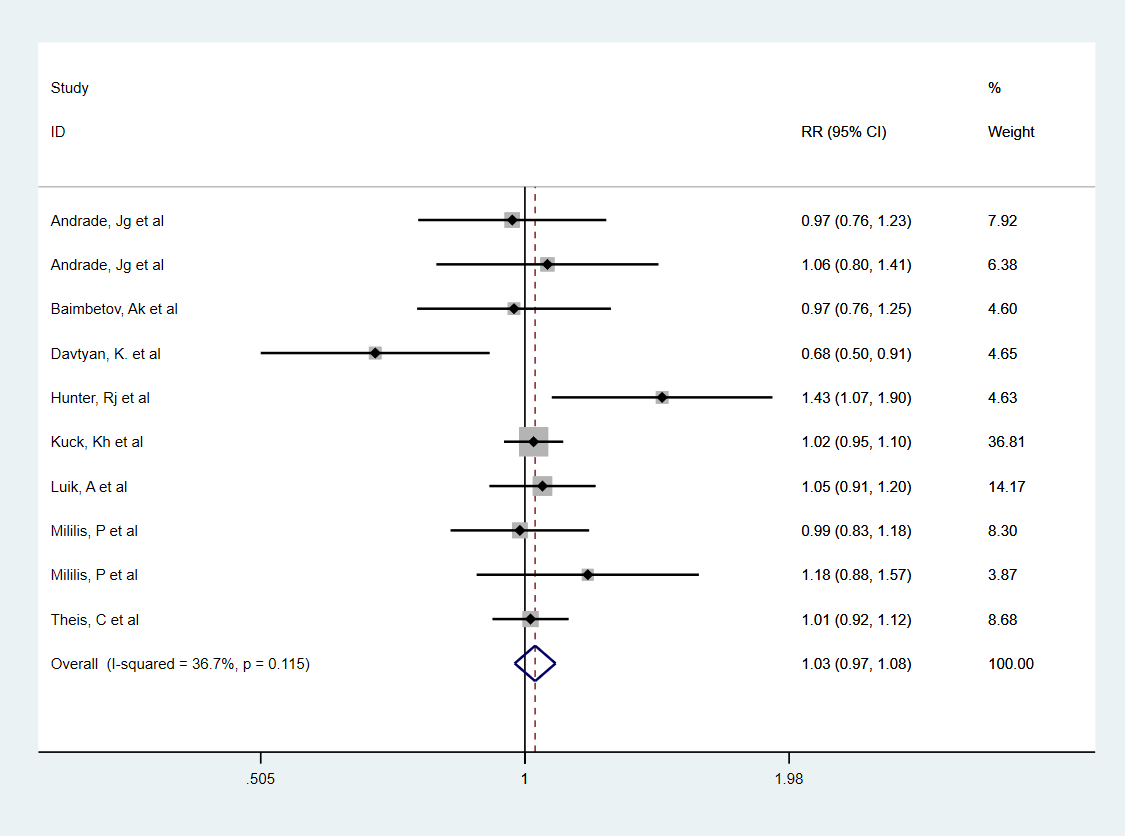


CBA VS RFA

*Note.* Subgroup analysis of randomized controlled trial (RCT)

RFA means Radiofrequency Ablation; CBA means Cryoballoon Ablation; RMN means Remote Magnetic Navigation Ablation; PFA means Pulsed Field Ablation

Fig. S25 **Inconsistency plot for PSM based on the outcome of freedom from AF and other AT**


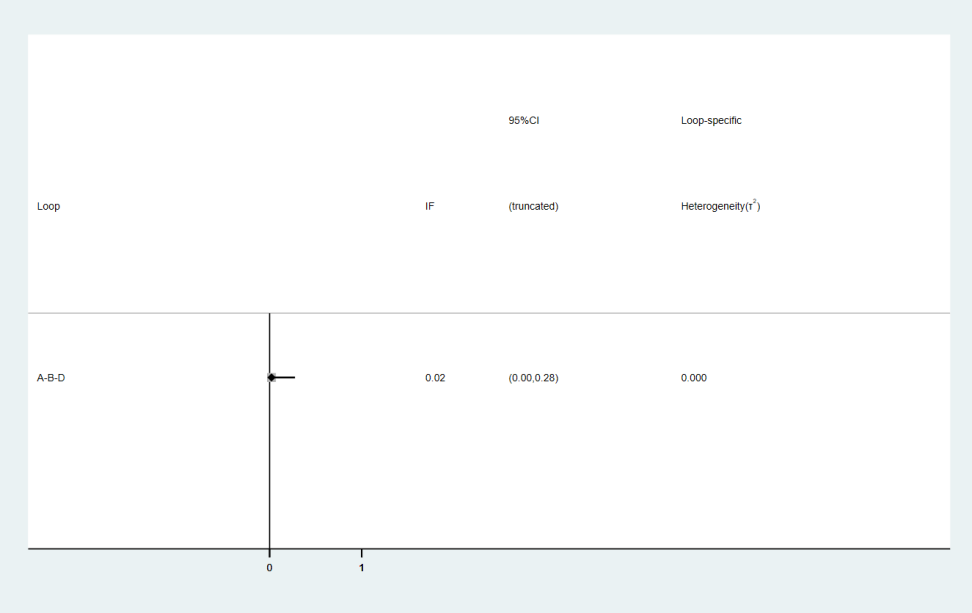


*Note.* A means Radiofrequency Ablation; B means Cryoballoon Ablation; D means Pulsed Field Ablation

Fig. S26 Subgroup analysis for different type of atrial fibrillation based on the outcome of complications


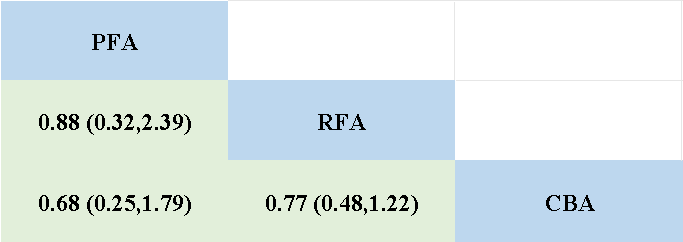


*Note.* Subgroup analysis of paroxysmal atrial fibrillation (PFA)


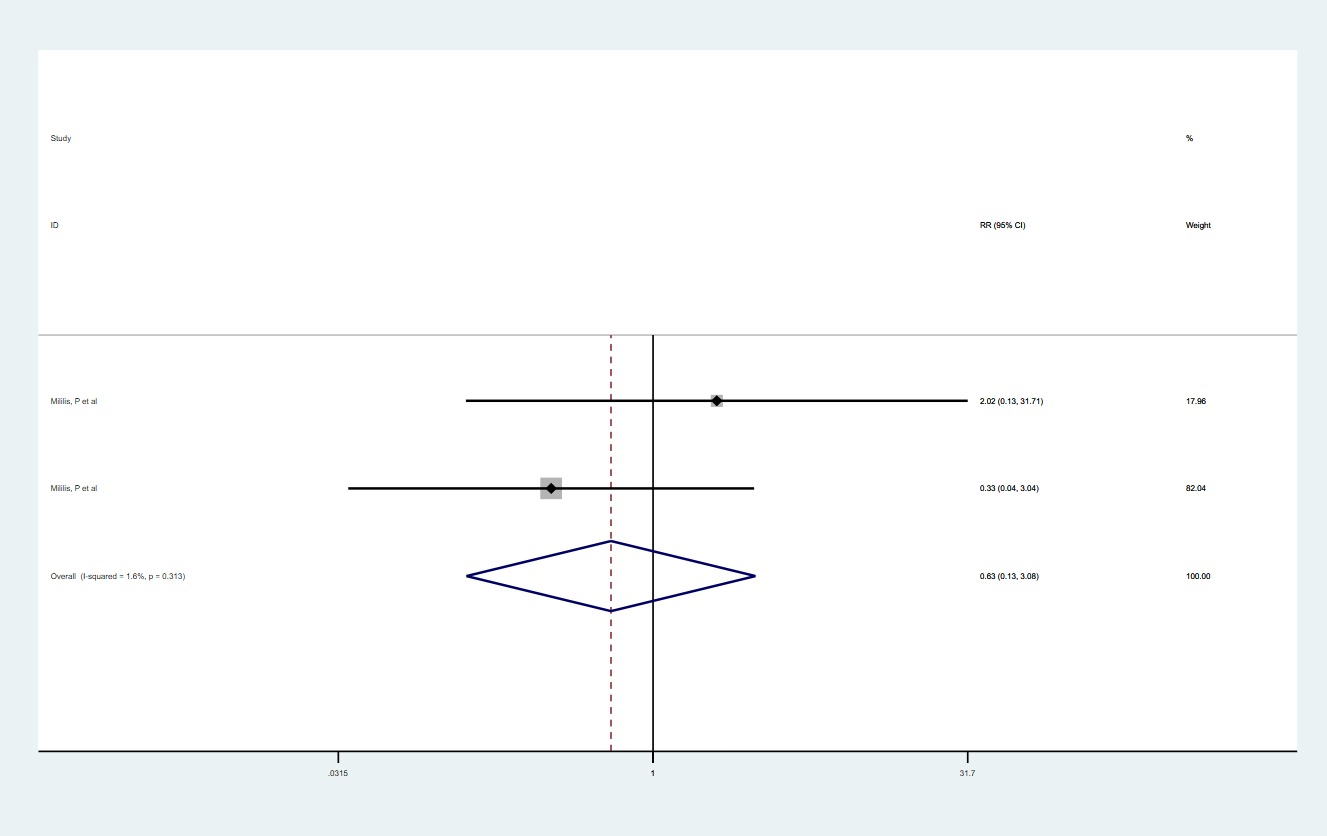


CBA VS RFA

*Note.* Subgroup analysis of persistent atrial fibrillation (PerAF)

RFA means Radiofrequency Ablation; CBA means Cryoballoon Ablation; PFA means Pulsed Field Ablation

Fig. S27 **Inconsistency plot for PAF based on the outcome of** complications

**
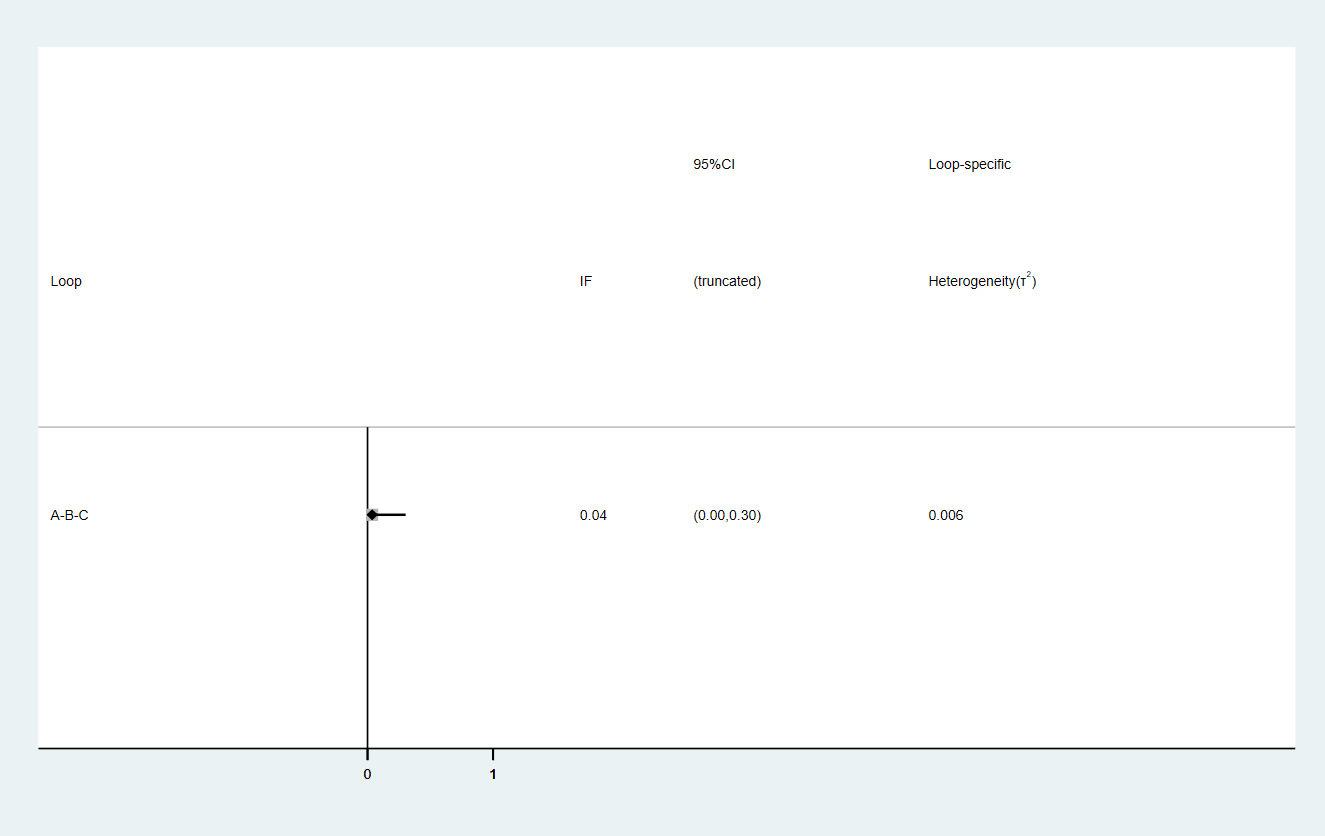
**

*Note. A* means Radiofrequency Ablation (RFA); B means Cryoballoon Ablation (CBA); C means Pulised Field Ablation (PFA)

Fig. S28 Subgroup analysis of different study design based on the outcome of complications.


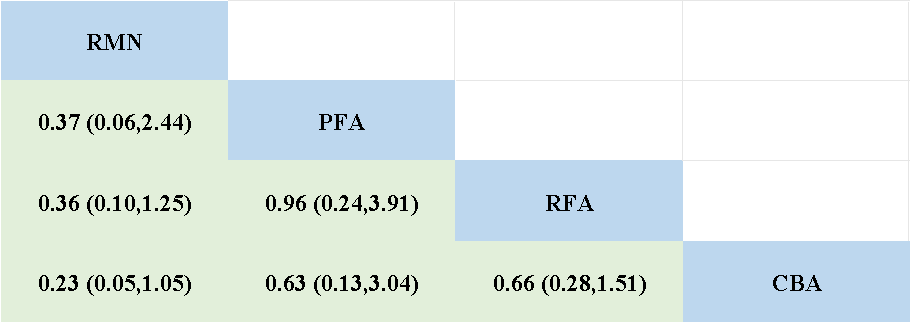


*Note.* Subgroup analysis of propensity-score matched studies (PSM)


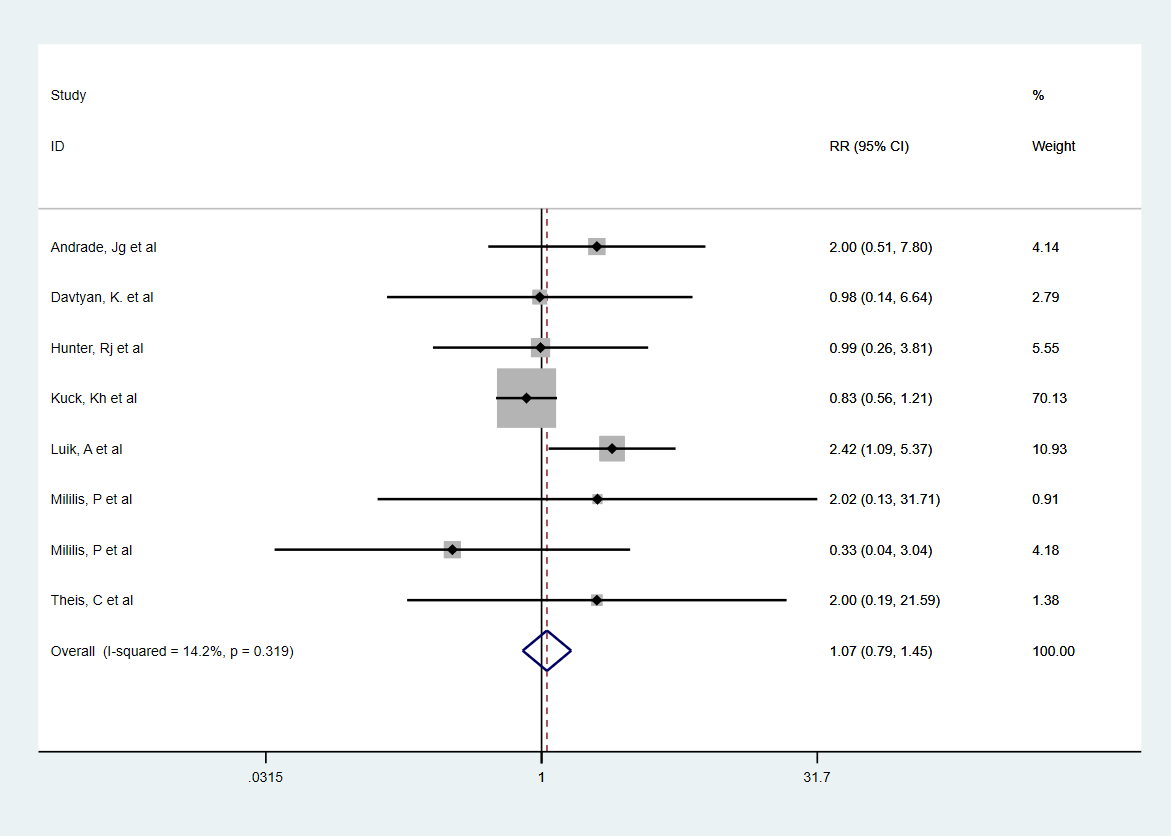


CBA VS RFA

*Note.* Subgroup analysis of randomized controlled trial (RCT)

RFA means Radiofrequency Ablation; CBA means Cryoballoon Ablation; RMN means Remote Magnetic Navigation Ablation; PFA means Pulsed Field Ablation

Fig. S29 **Inconsistency plot for PSM based on the outcome of** complications


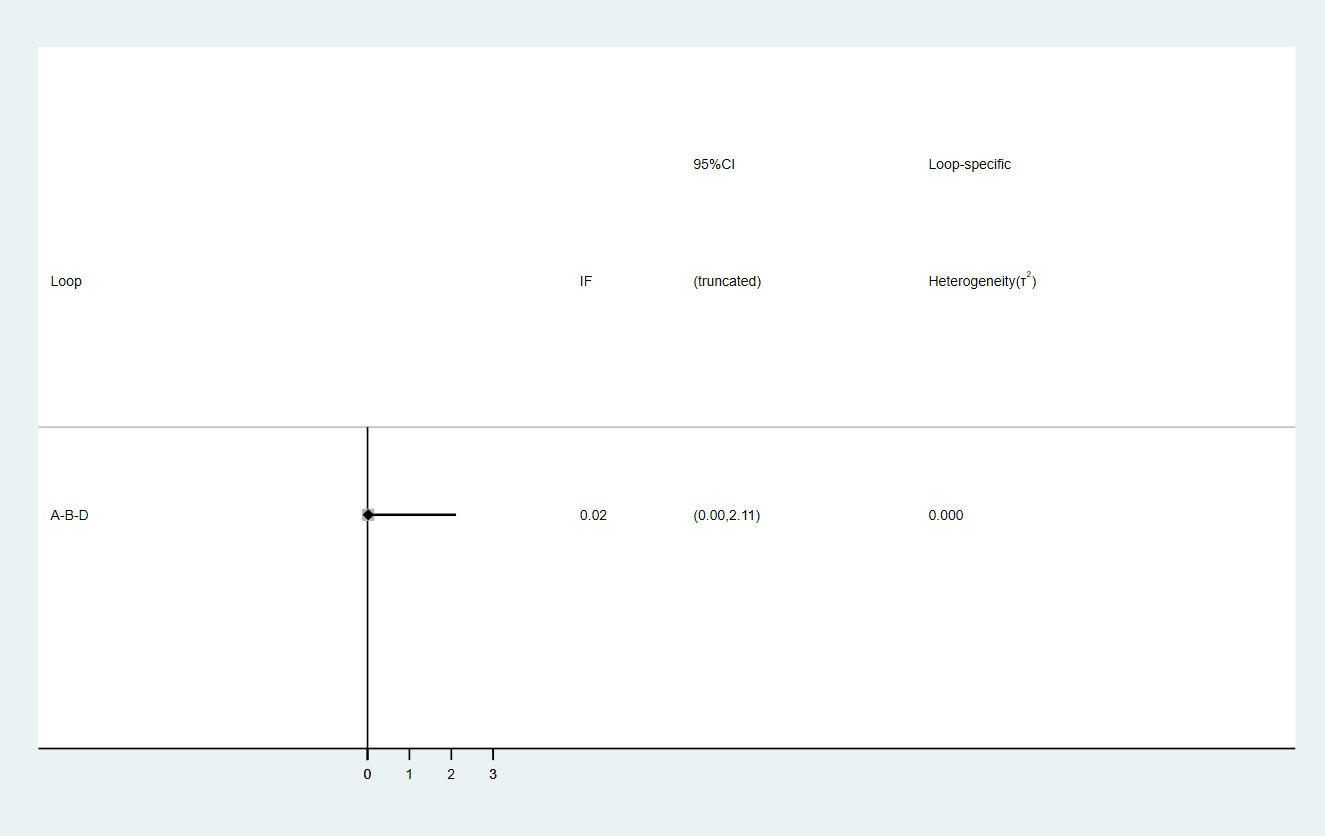


*Note.* A means Radiofrequency Ablation; B means Cryoballoon Ablation; D means Pulsed Field Ablation

Fig. S30 Subgroup analysis for different type of atrial fibrillation based on the outcome of procedure duration


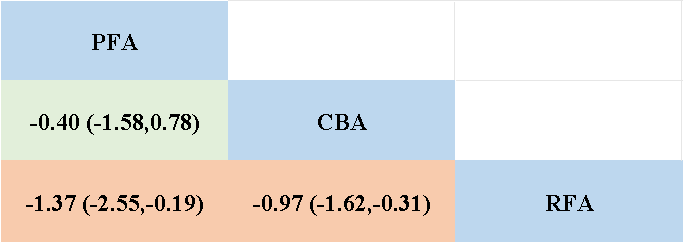


*Note.* Subgroup analysis of paroxysmal atrial fibrillation (PFA)


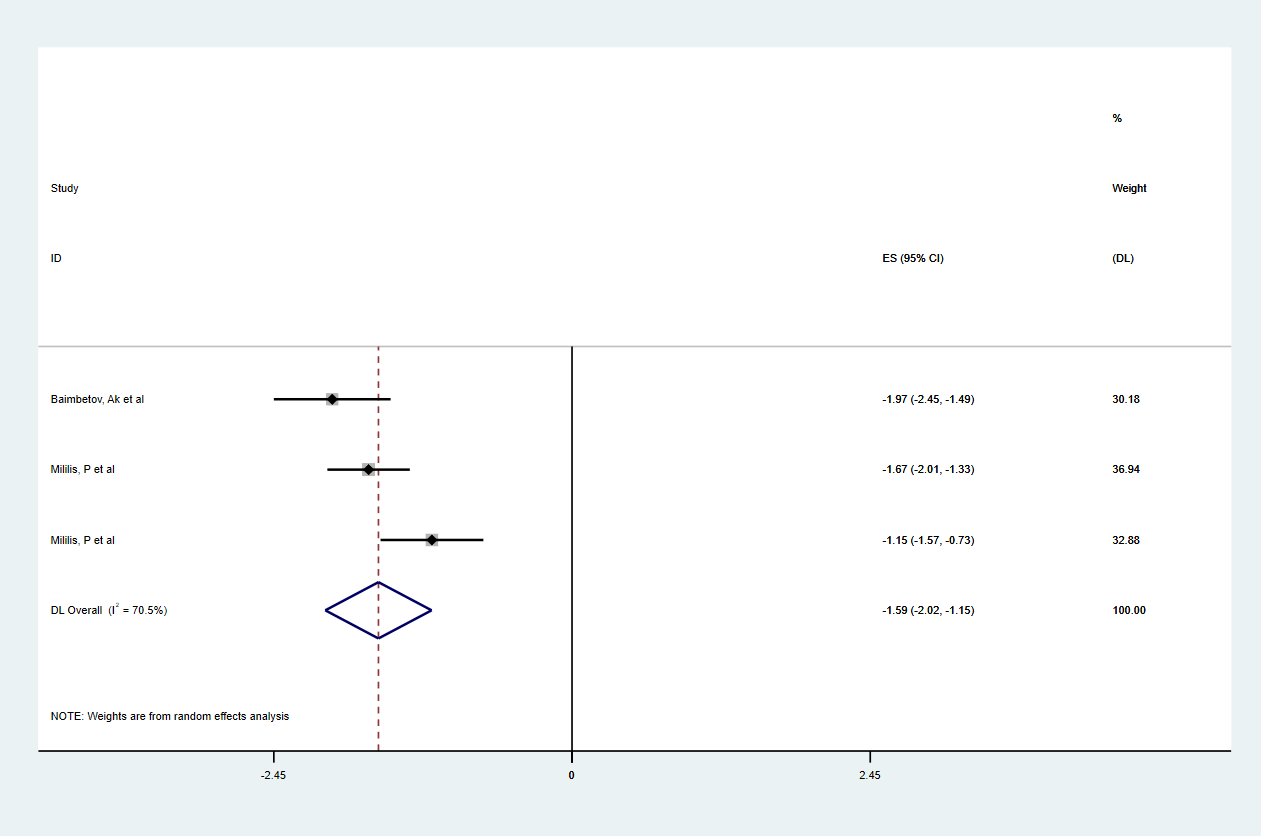


CBA VS RFA

*Note.* Subgroup analysis of persistent atrial fibrillation (PerAF)

RFA means Radiofrequency Ablation; CBA means Cryoballoon Ablation; PFA means Pulsed Field Ablation

Fig. S31 **Inconsistency plot for PAF based on the outcome of** procedure duration

**
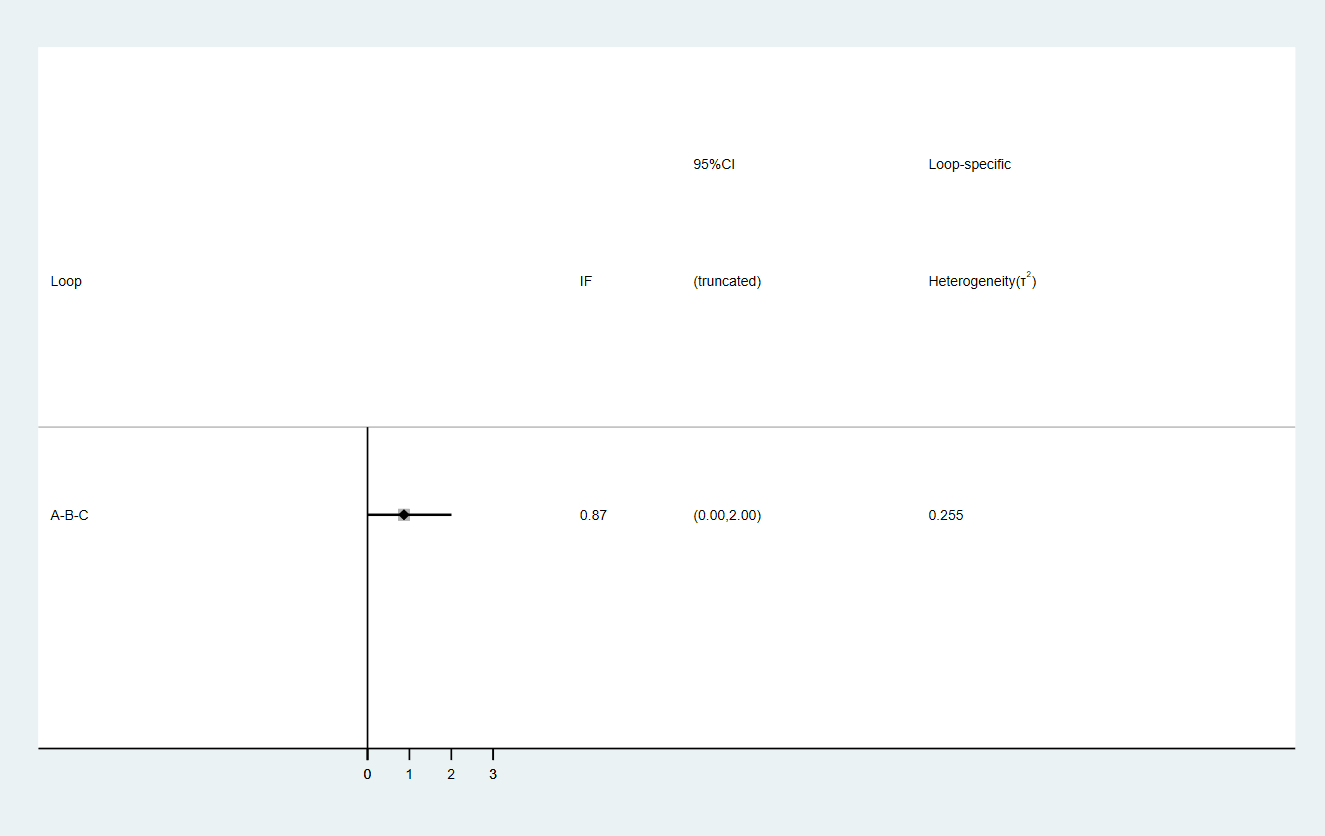
**

*Note. A* means Radiofrequency Ablation (RFA); B means Cryoballoon Ablation (CBA); C means Pulised Field Ablation (PFA)

Fig. S32 Subgroup analysis of different study design based on the outcome of procedure duration


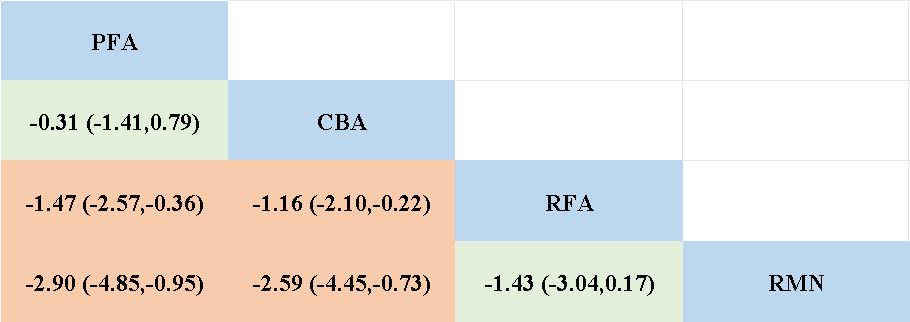


*Note.* Subgroup analysis of propensity-score matched studies (PSM)


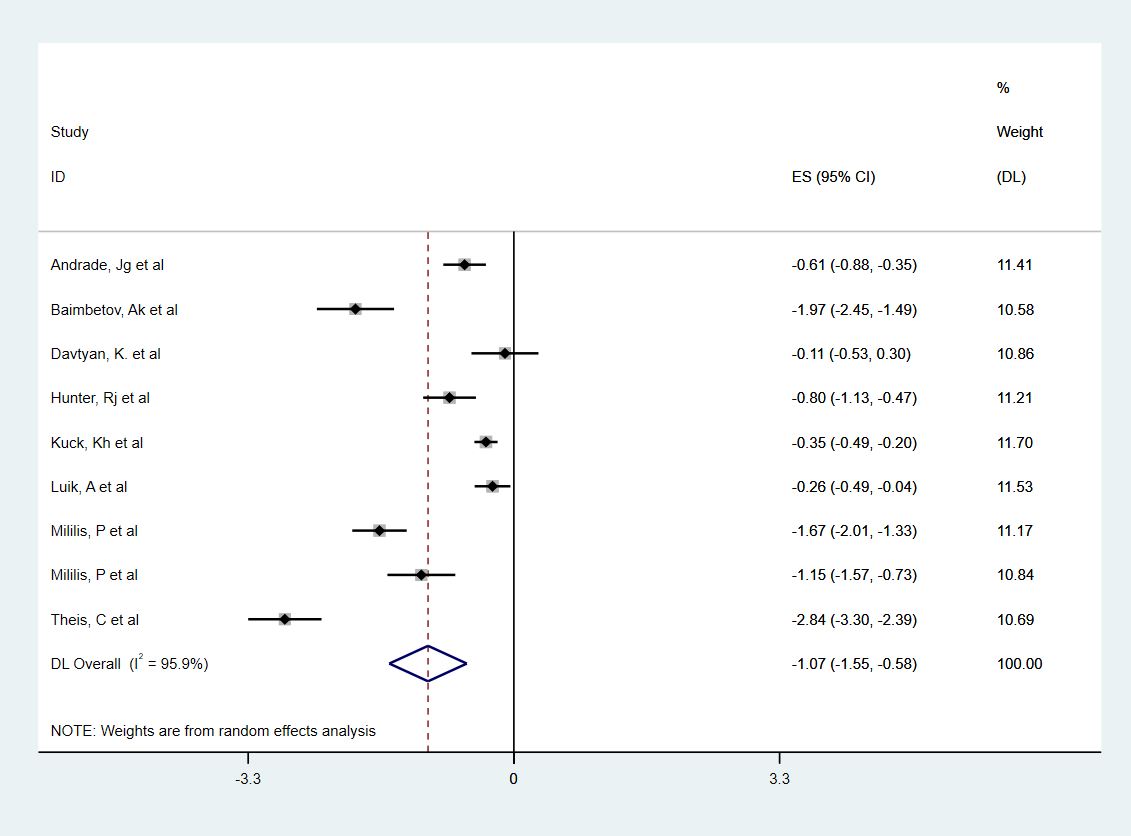


CBA VS RFA

*Note.* Subgroup analysis of randomized controlled trial (RCT)

RFA means Radiofrequency Ablation; CBA means Cryoballoon Ablation; RMN means Remote Magnetic Navigation Ablation; PFA means Pulsed Field Ablation

Fig. S33 **Inconsistency plot for PSM based on the outcome of** procedure duration


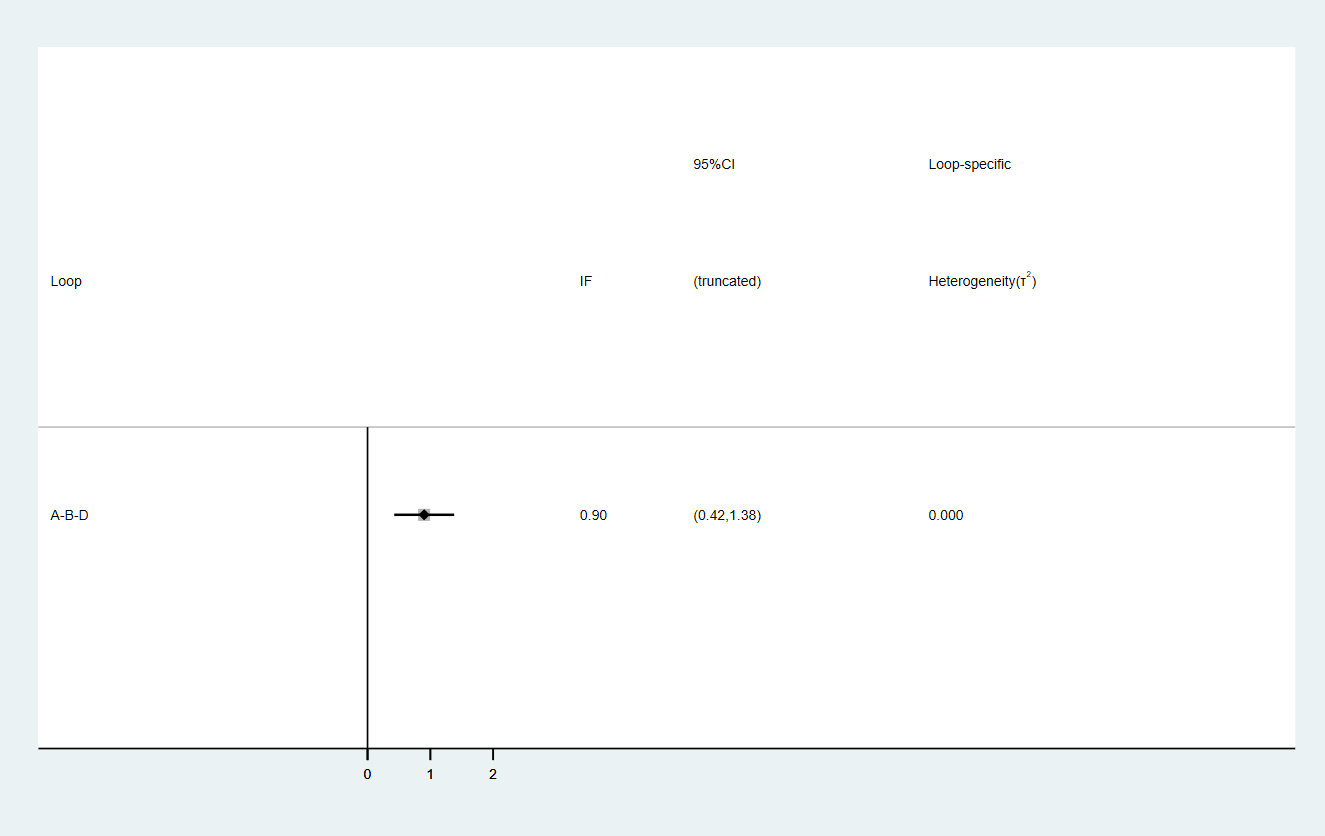


*Note.* A means Radiofrequency Ablation; B means Cryoballoon Ablation; D means Pulsed Field Ablation

Fig. S34 Subgroup analysis for different type of atrial fibrillation based on the outcome of fluoroscopy duration


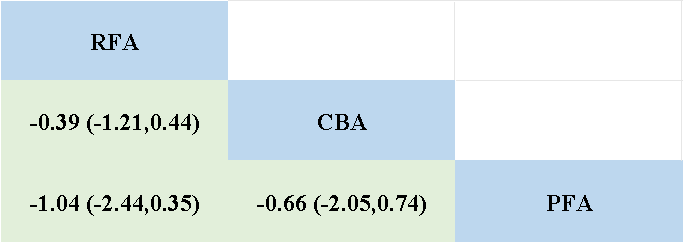


*Note.* Subgroup analysis of paroxysmal atrial fibrillation (PFA)


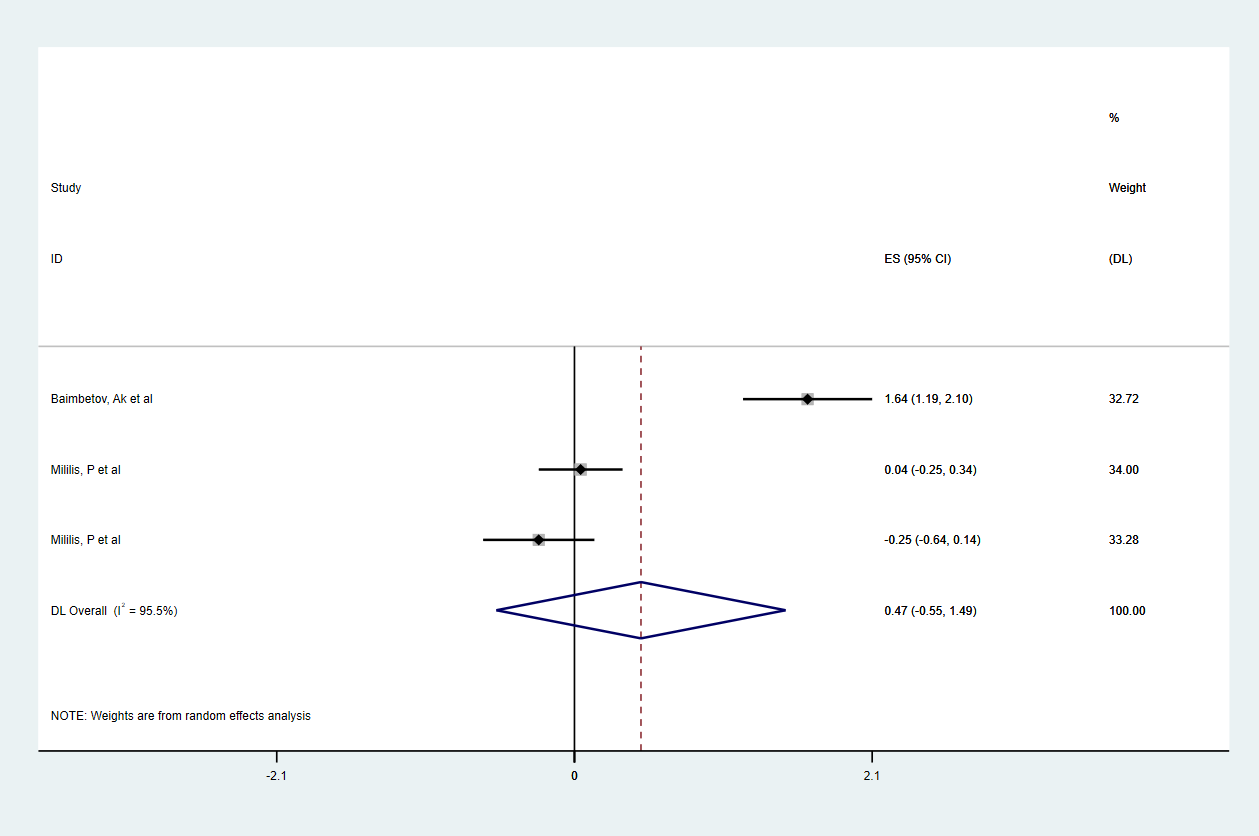


CBA VS RFA

*Note.* Subgroup analysis of persistent atrial fibrillation (PerAF)

RFA means Radiofrequency Ablation; CBA means Cryoballoon Ablation; PFA means Pulsed Field Ablation

Fig. S35 **Inconsistency plot for PAF based on the outcome of** fluoroscopy duration

**
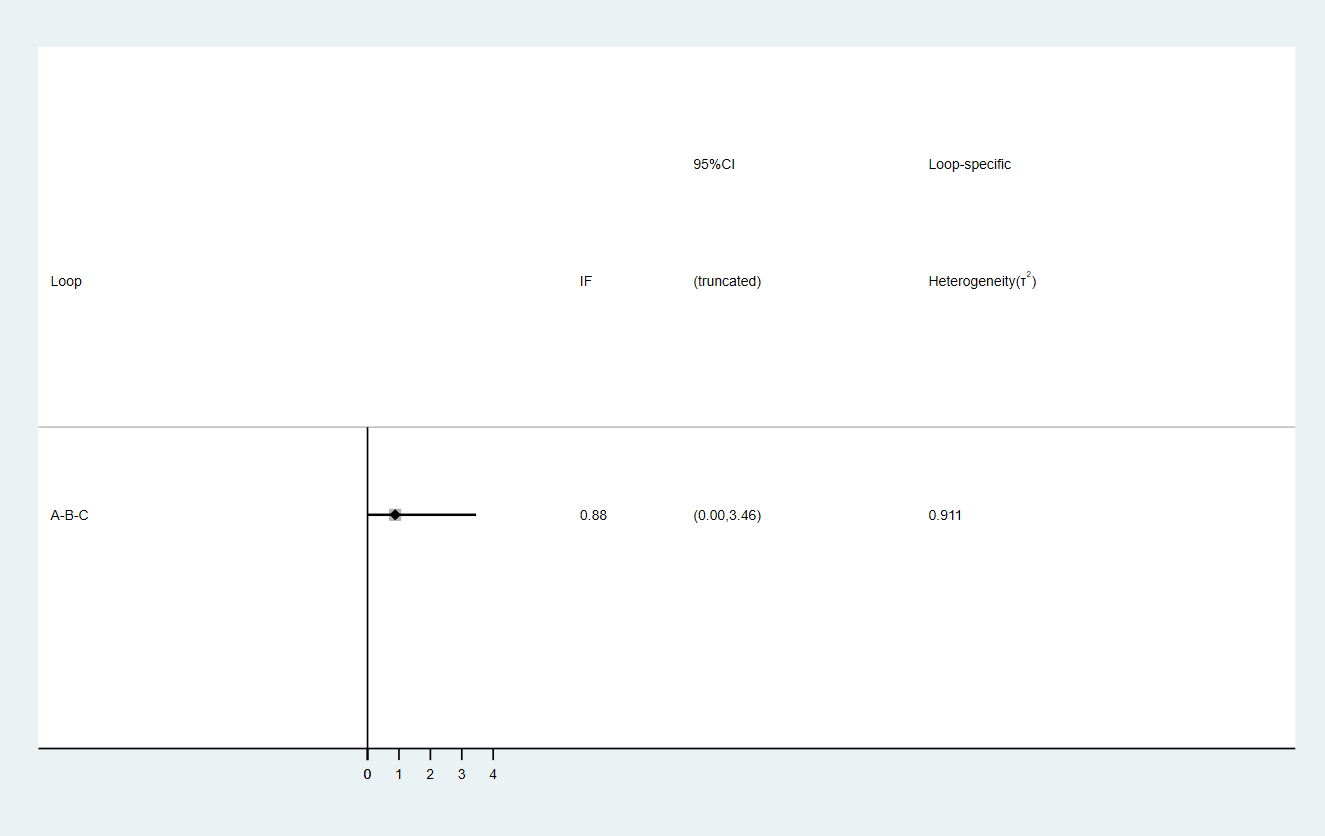
**

*Note. A* means Radiofrequency Ablation (RFA); B means Cryoballoon Ablation (CBA); C means Pulised Field Ablation (PFA)

Fig. S36 Subgroup analysis of different study design based on the outcome of fluoroscopy duration


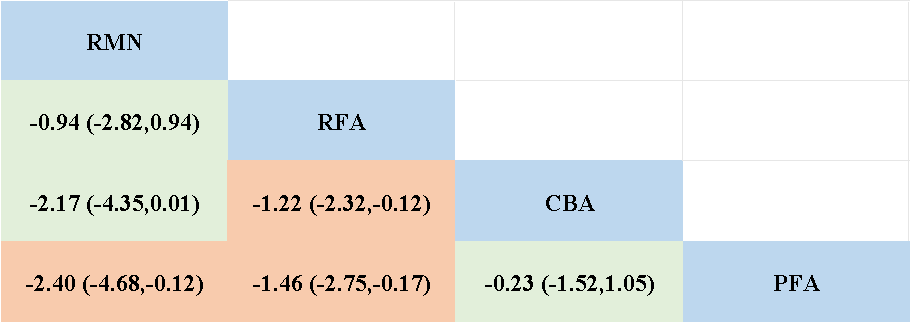


*Note.* Subgroup analysis of propensity-score matched studies (PSM)


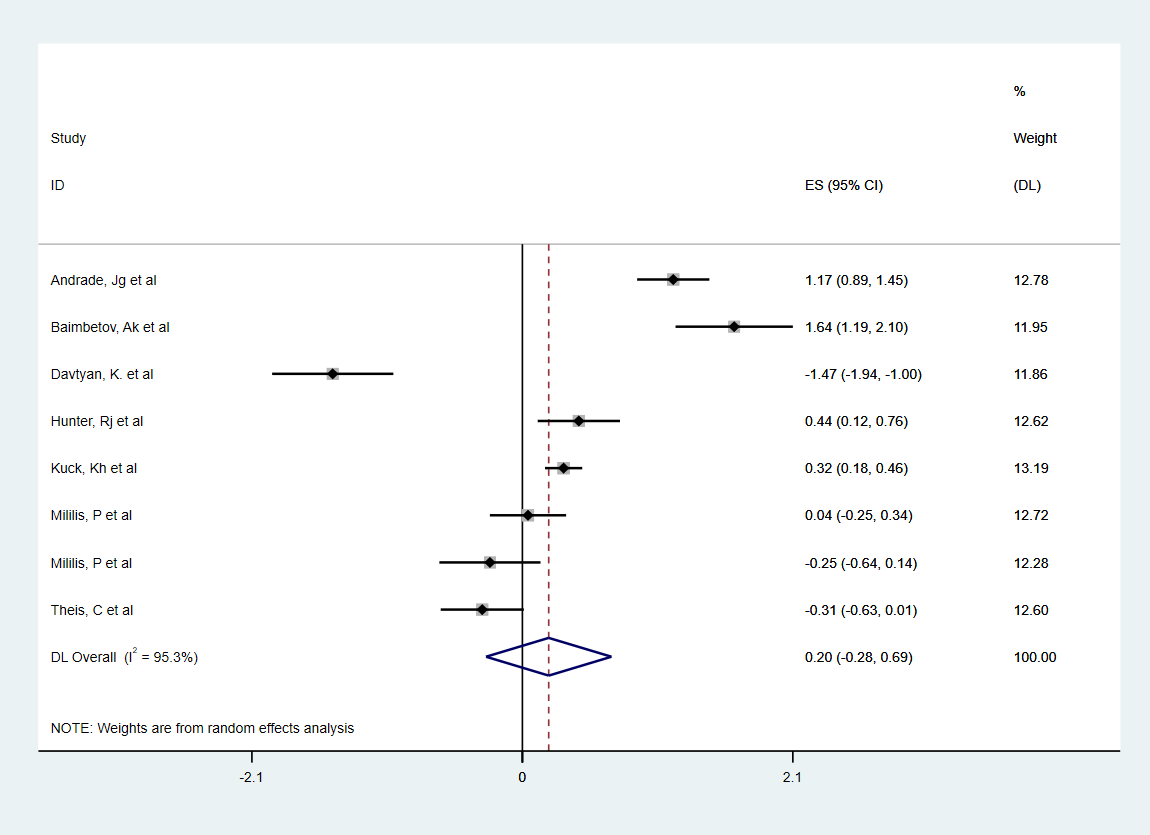


CBA VS RFA

*Note.* Subgroup analysis of randomized controlled trial (RCT)

RFA means Radiofrequency Ablation; CBA means Cryoballoon Ablation; RMN means Remote Magnetic Navigation Ablation; PFA means Pulsed Field Ablation

Fig. S37 **Inconsistency plot for PSM based on the outcome of** fluoroscopy duration


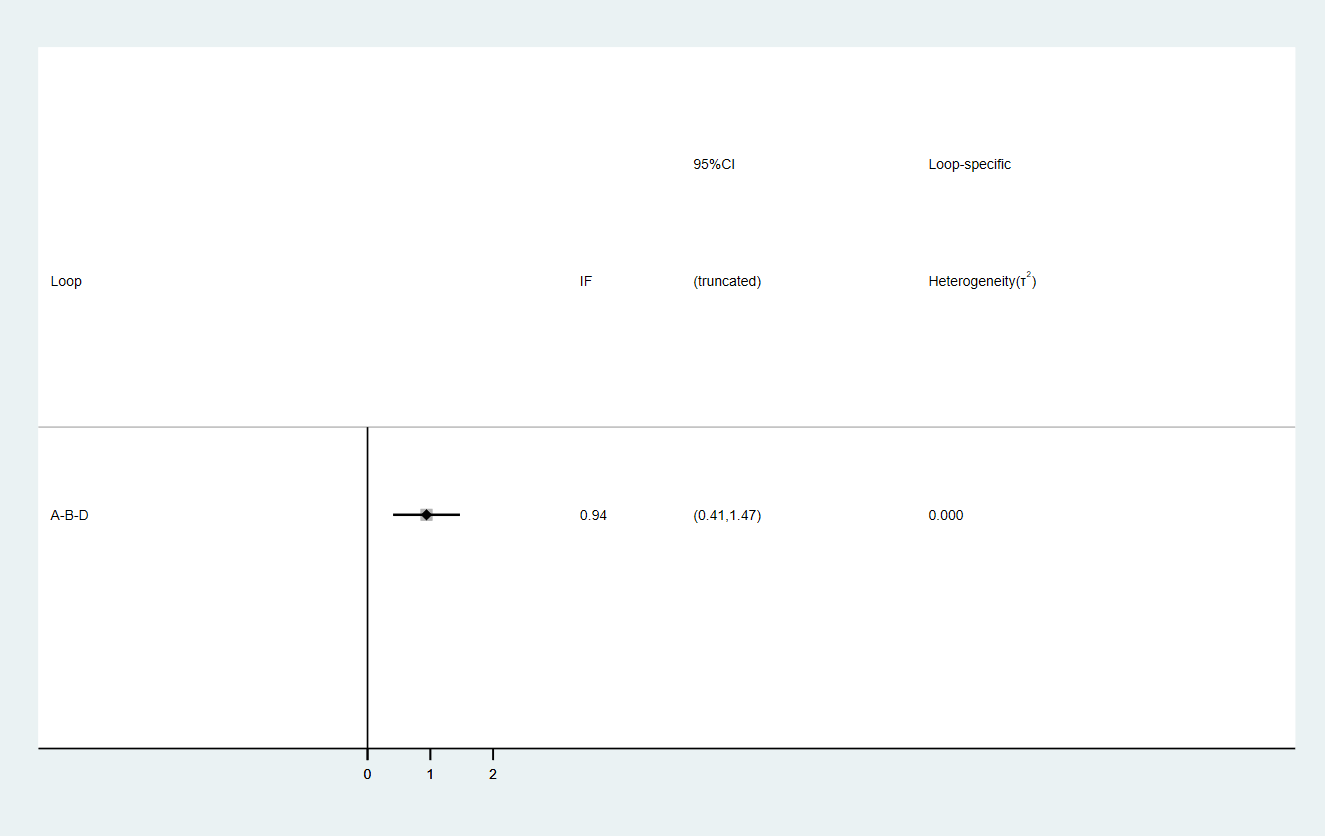


*Note.* A means Radiofrequency Ablation; B means Cryoballoon Ablation; D means Pulsed Field Ablation

S8. Sensitivity analysis

Fig. S38 Network evidence of sensitivity analysis based on the outcome of freedom from AF and other AT (12 months)


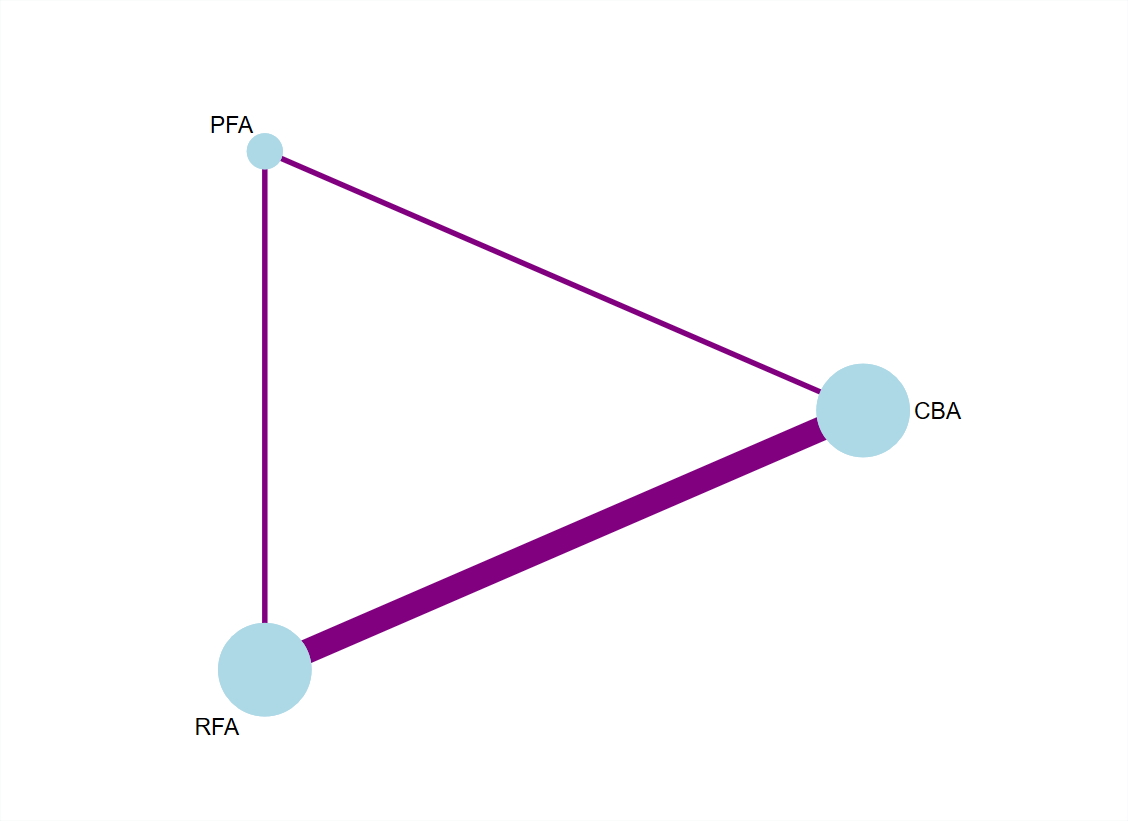


Figure. S39 SUCRA plot of sensitivity analysis based on the freedom from AF and other AT outcome (12 months)


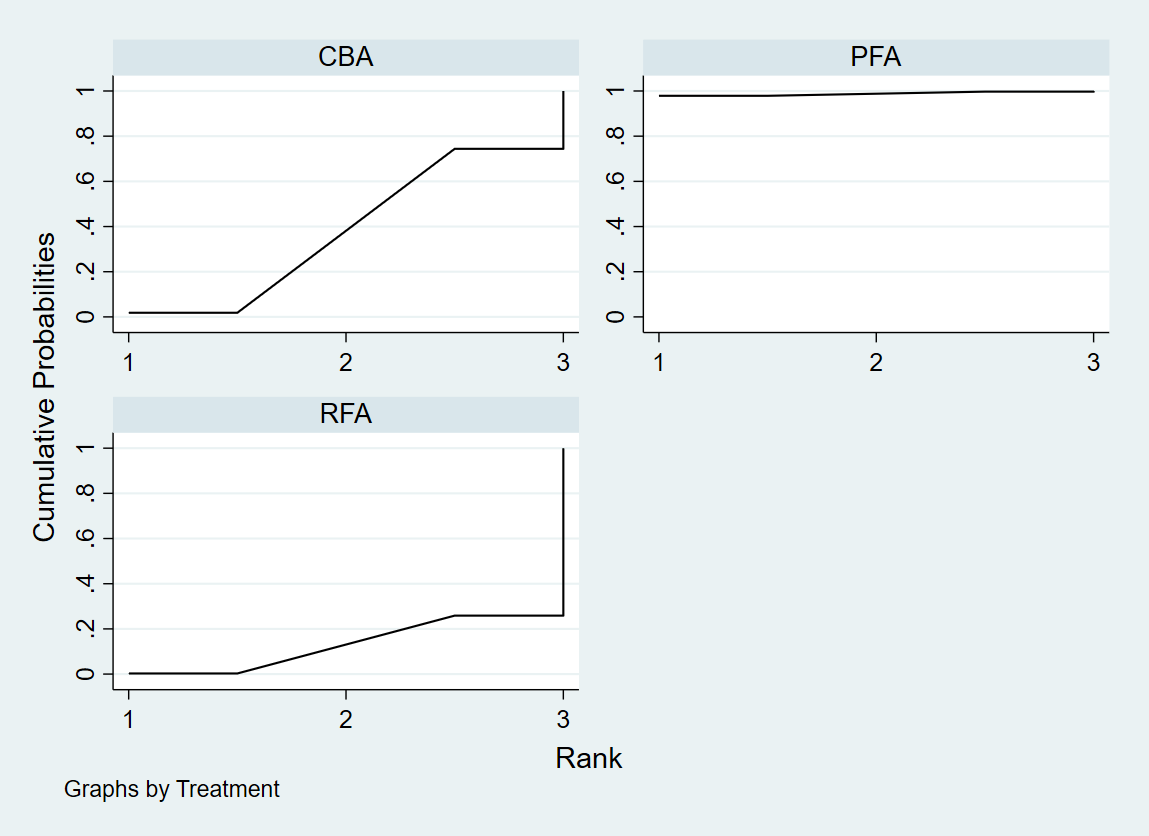


*Note.* RFA means Radiofrequency Ablation; CBA means Cryoballoon Ablation; PFA means Pulsed Field Ablation

Figure. S40 League table of sensitivity analysis based on the outcome of freedom from AF and other AT (12 months)


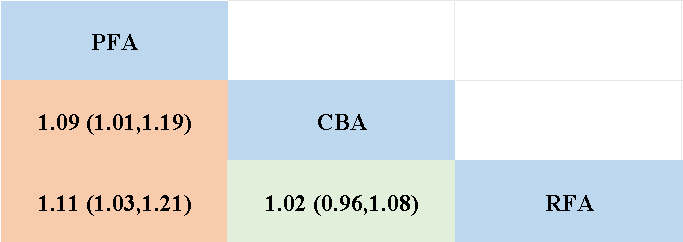


*Note.* RFA means Radiofrequency Ablation; CBA means Cryoballoon Ablation; PFA means Pulsed Field Ablation

Figure. S41 Funnel plot of sensitivity analysis based on the freedom from AF and other AT outcome (12 months)


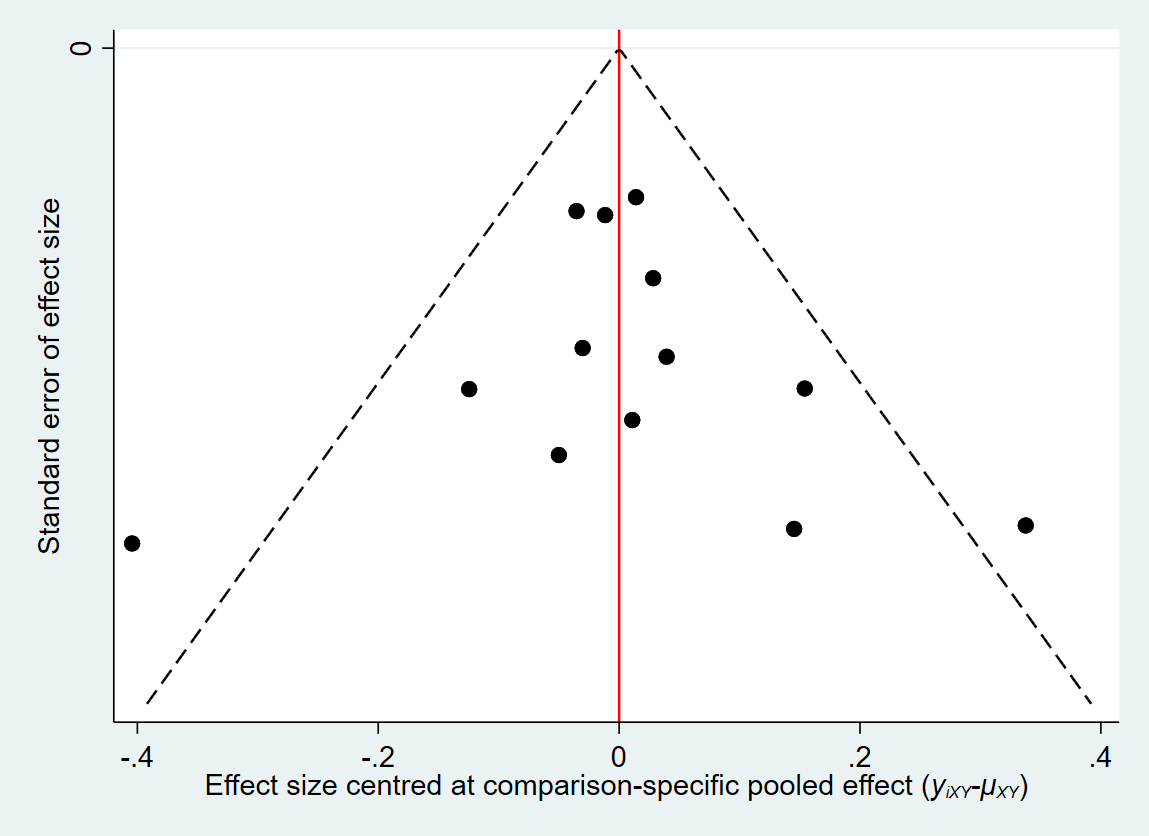


Fig. S42 Network evidence of sensitivity analysis based on the outcome of complications (12 months)


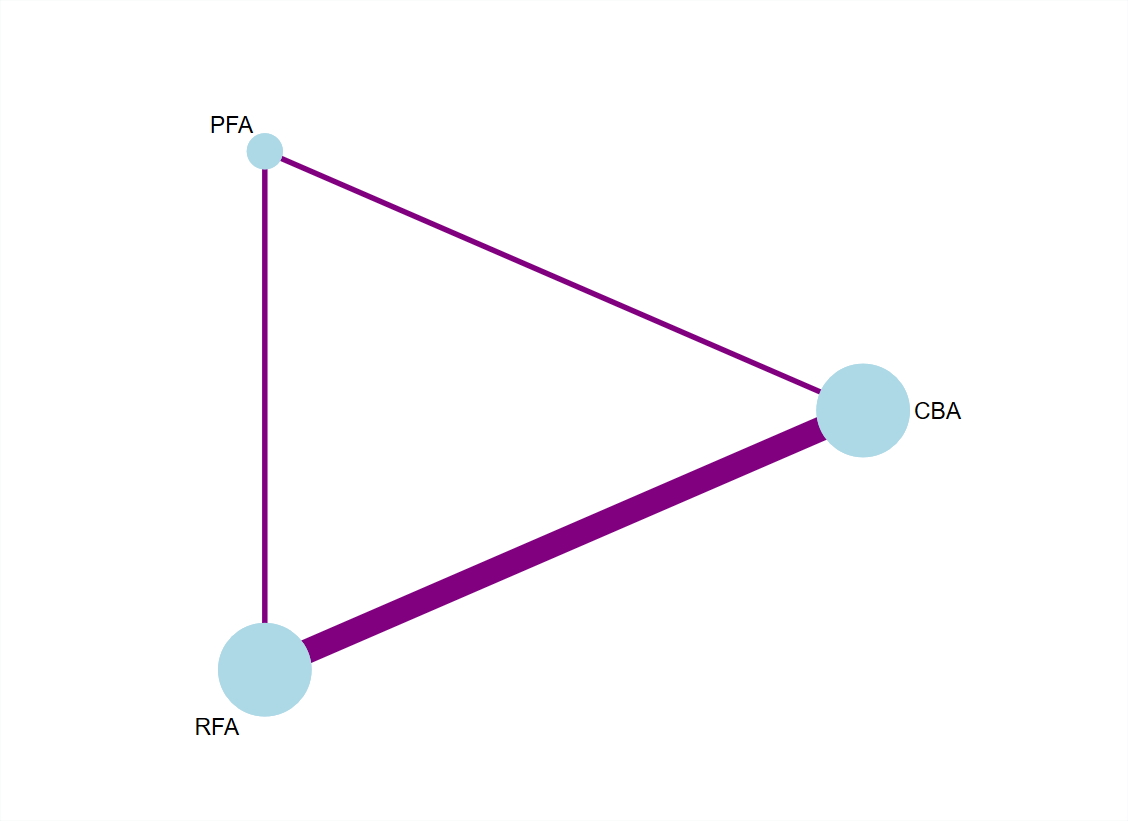


Figure. S43 SUCRA plot of sensitivity analysis based on the complications outcome (12 months)


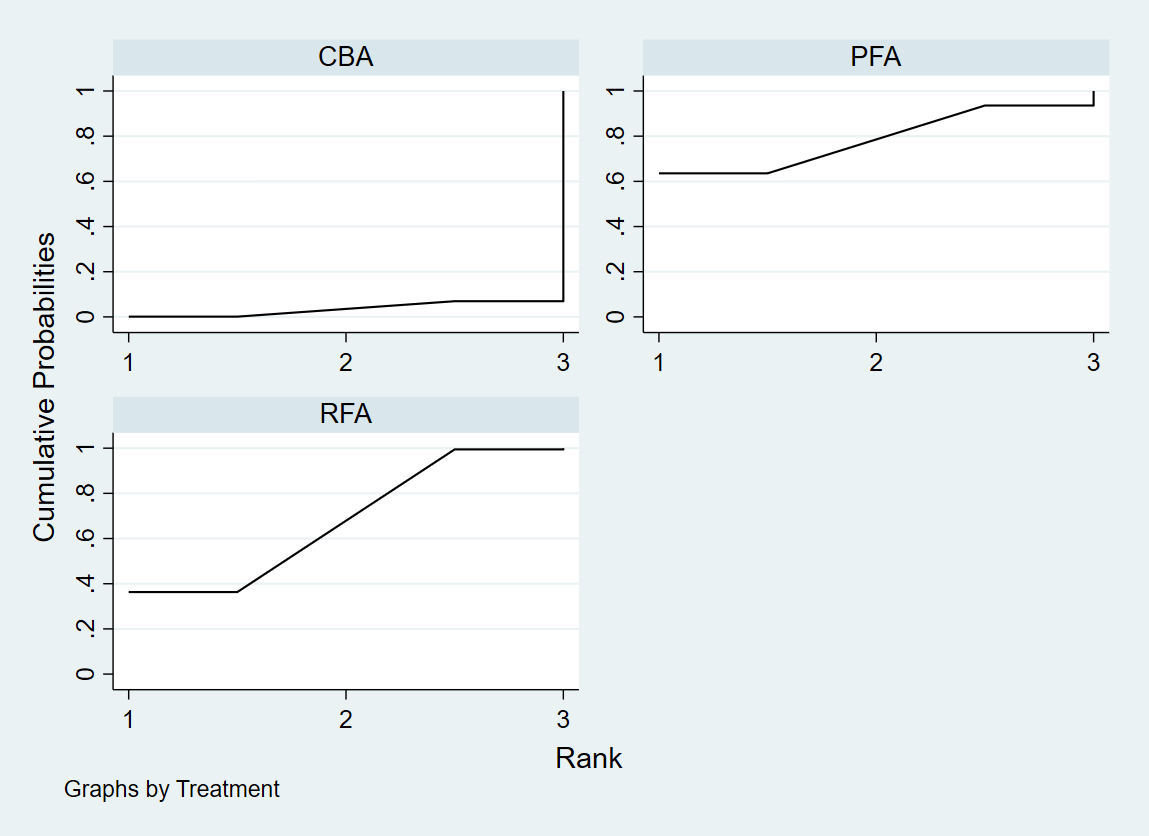


*Note.* RFA means Radiofrequency Ablation; CBA means Cryoballoon Ablation; PFA means Pulsed Field Ablation

Figure. S44 League table of sensitivity analysis based on the outcome of complications (12 months)


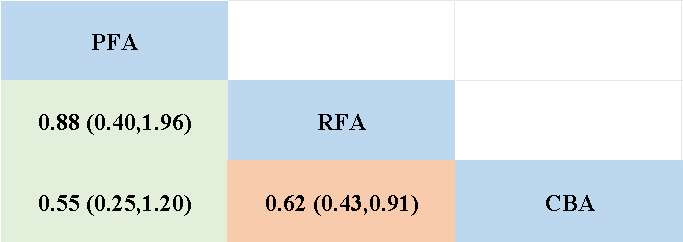


*Note.* RFA means Radiofrequency Ablation; CBA means Cryoballoon Ablation; PFA means Pulsed Field Ablation

Figure. S45 Funnel plot of sensitivity analysis based on the complications outcome (12 months)


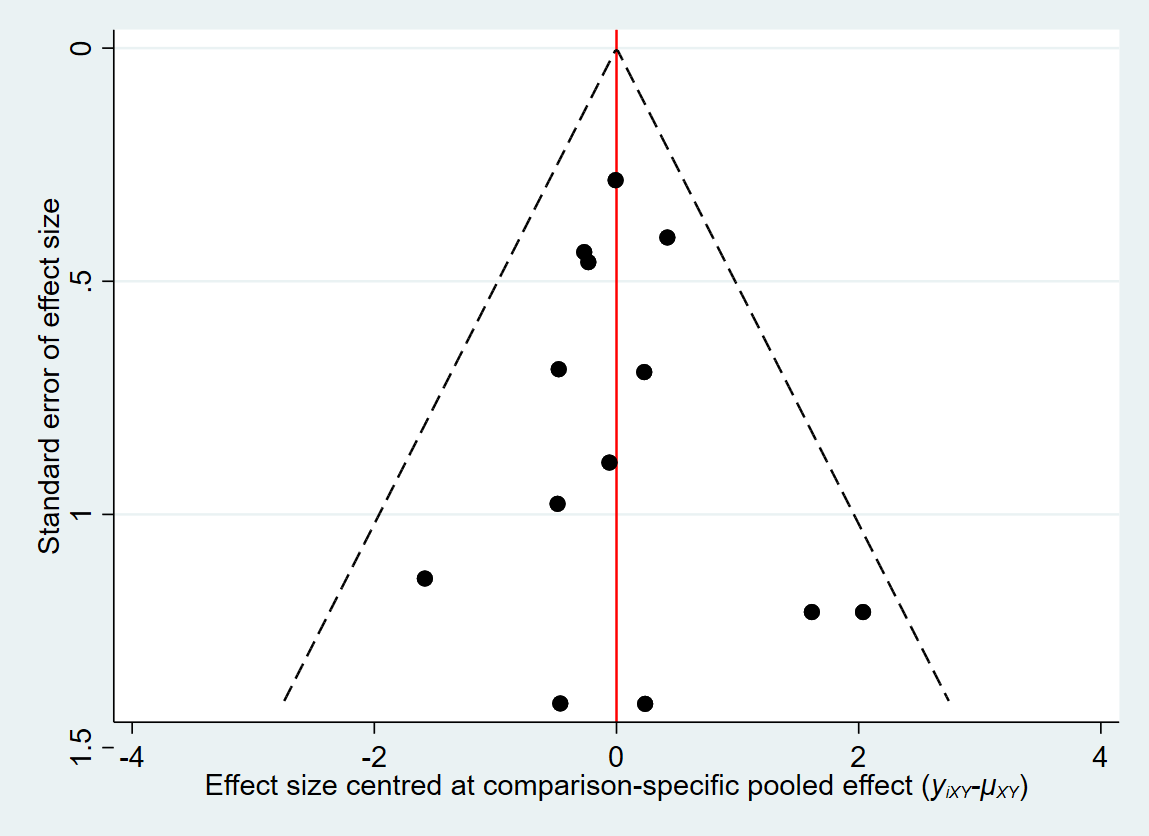


Fig. S46 Network evidence of sensitivity analysis based on the outcome of procedure duration (12 months)


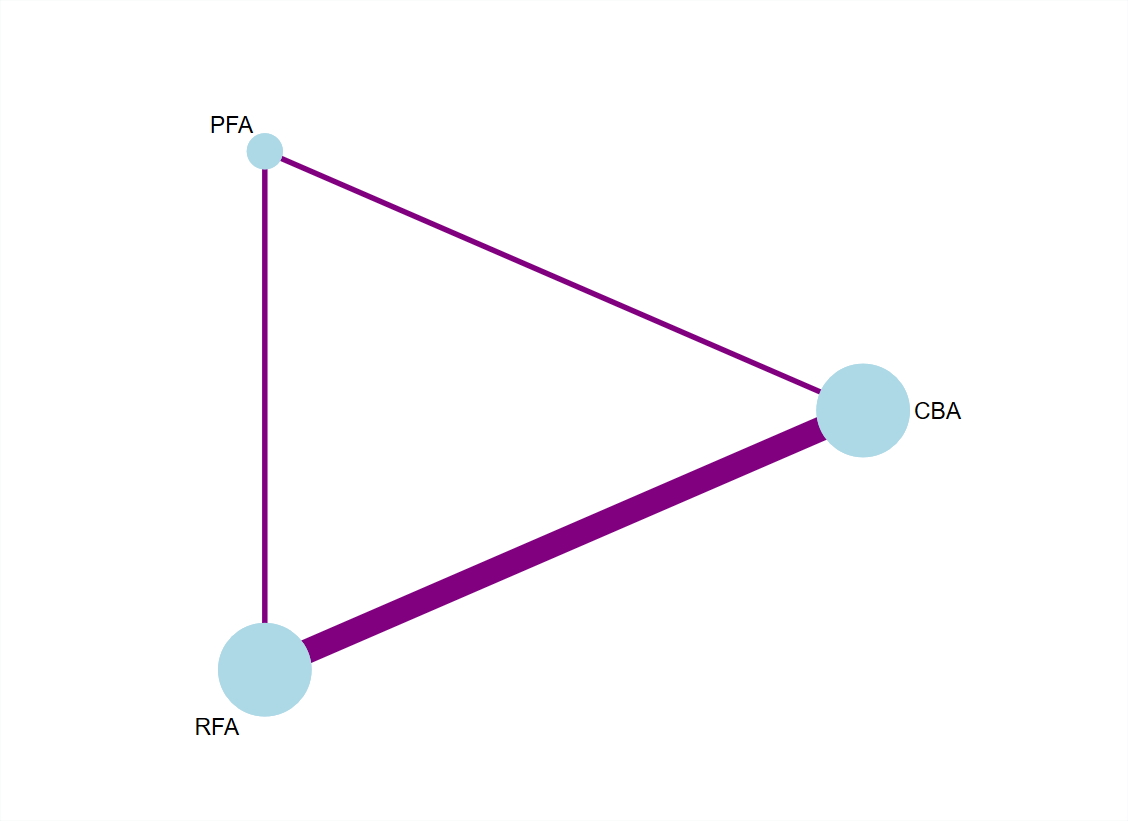


Figure. S47 SUCRA plot of sensitivity analysis based on the procedure duration outcome (12 months)


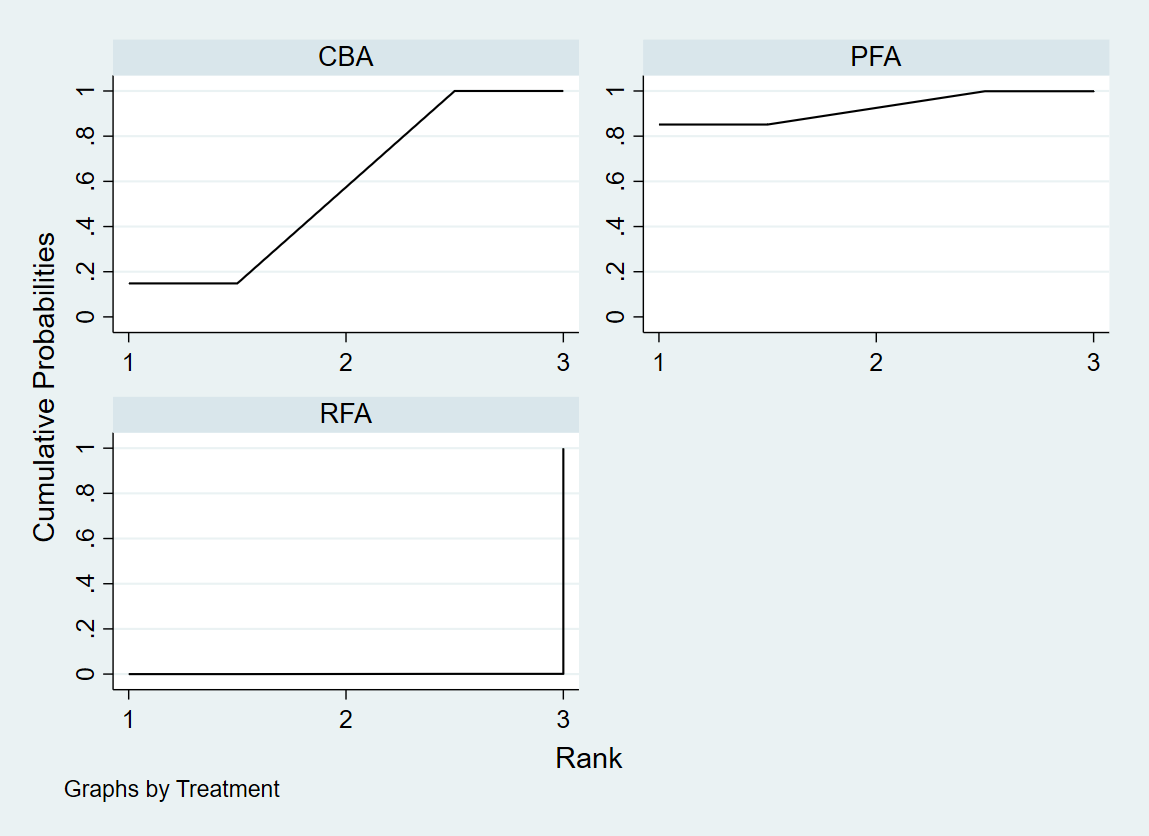


*Note.* RFA means Radiofrequency Ablation; CBA means Cryoballoon Ablation; PFA means Pulsed Field Ablation

Figure. S48 League table of sensitivity analysis based on the outcome of procedure duration (12 months)


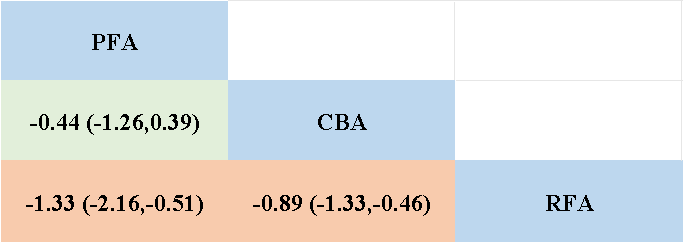


*Note.* RFA means Radiofrequency Ablation; CBA means Cryoballoon Ablation; PFA means Pulsed Field Ablation

Figure. S49 Funnel plot of sensitivity analysis based on the procedure duration outcome (12 months)


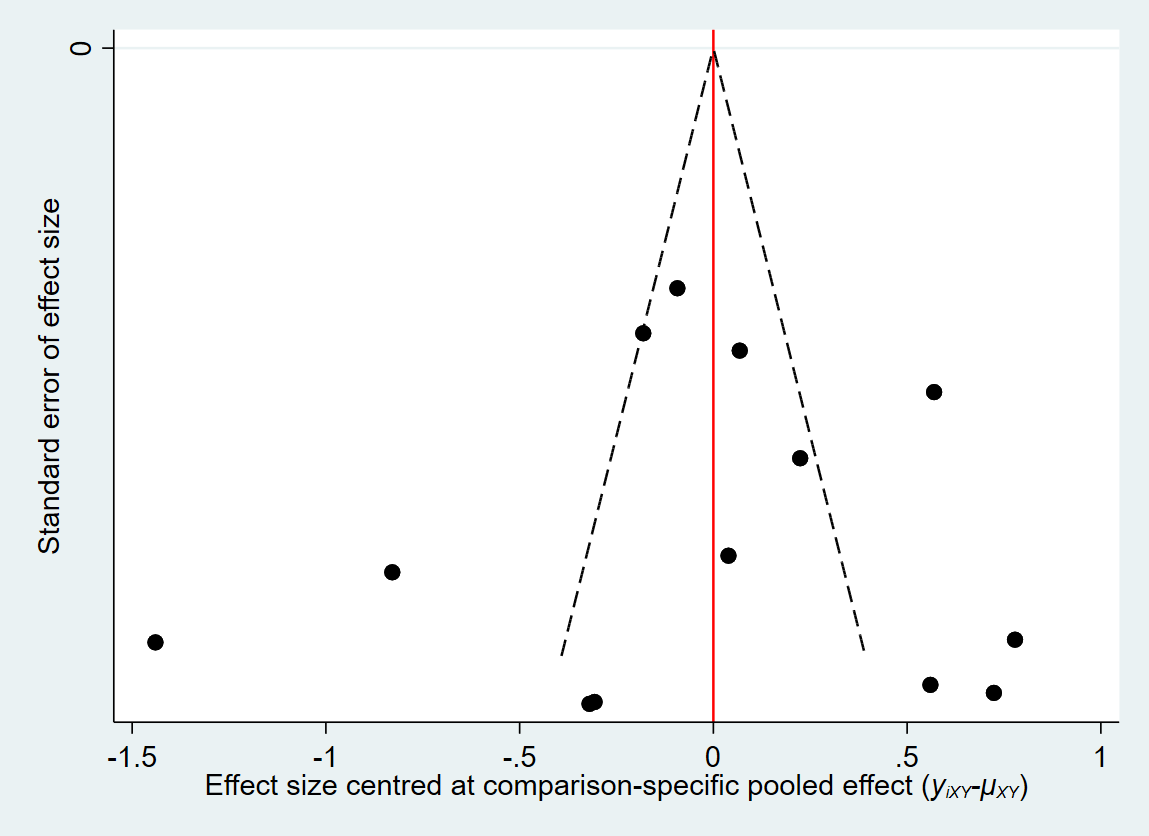


Fig. S50 Network evidence of sensitivity analysis based on the outcome of fluoroscopy duration (12 months)


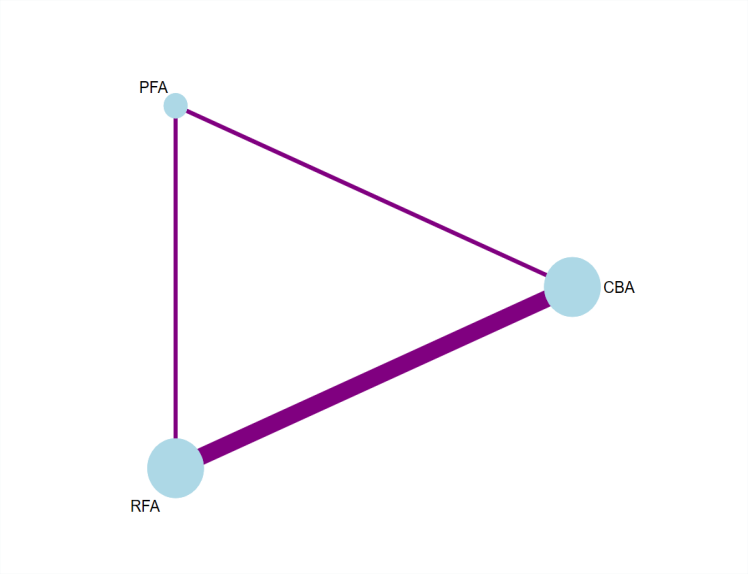


Figure. S51 SUCRA plot of sensitivity analysis based on the fluoroscopy duration outcome (12 months)


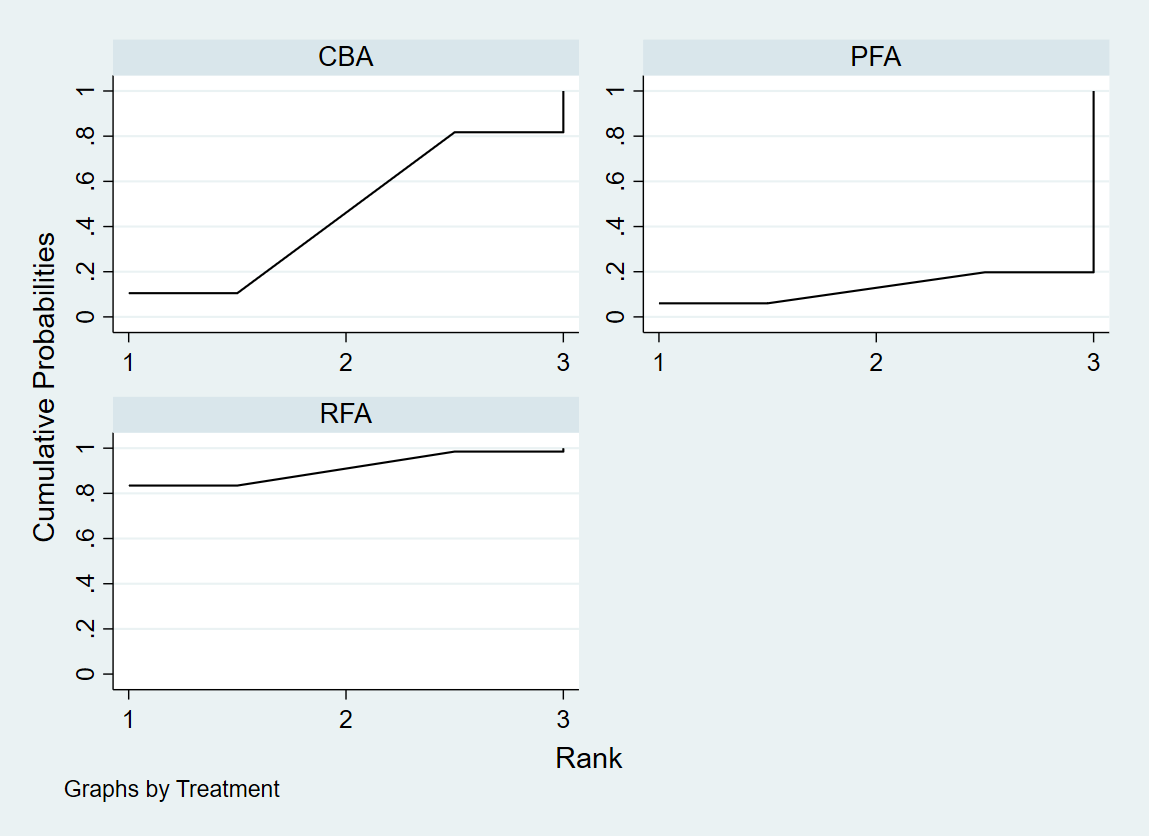


*Note.* RFA means Radiofrequency Ablation; CBA means Cryoballoon Ablation; PFA means Pulsed Field Ablation

Figure. S52 League table of sensitivity analysis based on the outcome of fluoroscopy duration (12 months)


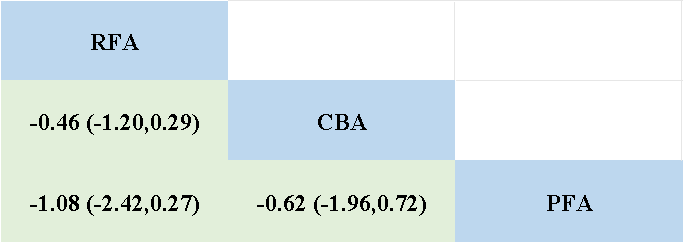


*Note.* RFA means Radiofrequency Ablation; CBA means Cryoballoon Ablation; PFA means Pulsed Field Ablation

Figure. S53 Funnel plot of sensitivity analysis based on the fluoroscopy duration outcome (12 months)


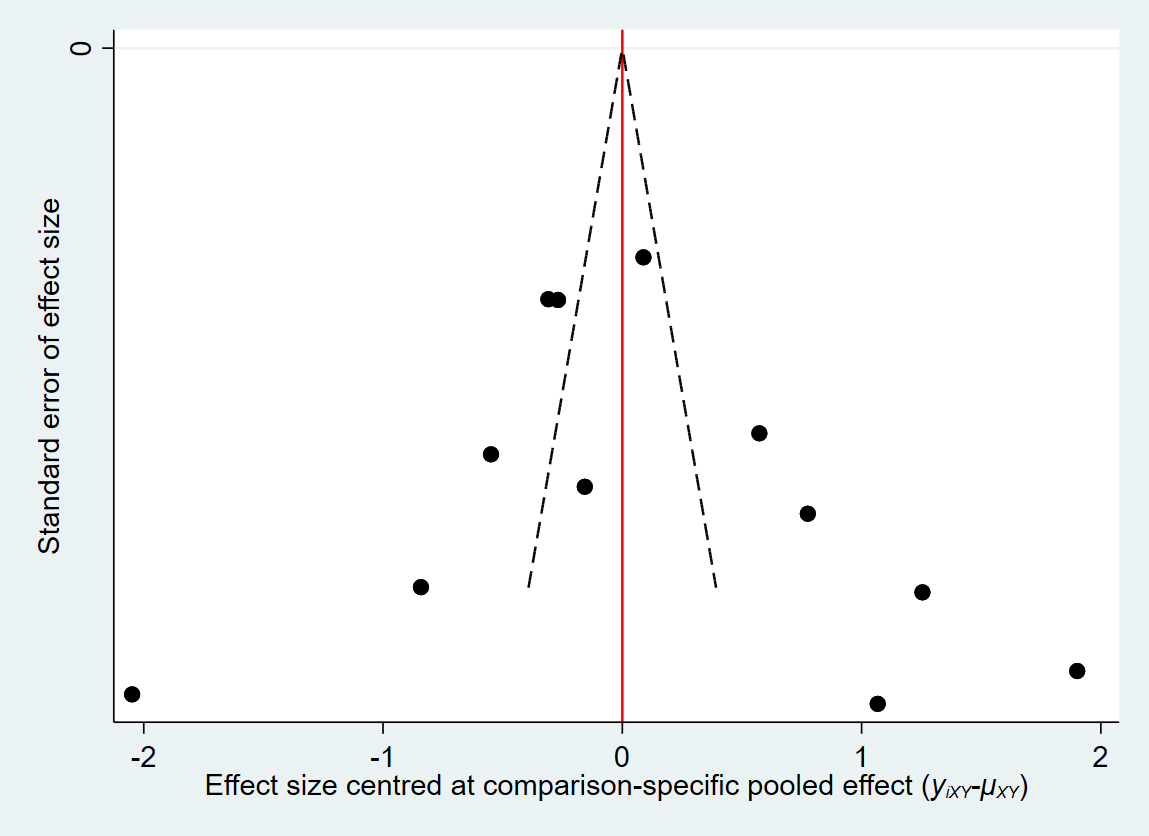


S9. Summary table of pairwise comparison results

Table. S2 Dierct comparison treatment effect with RFA

| Outcomes | Types | Trials | Effect size | I² |
| --- | --- | --- | --- | --- |
| Freedom from AF and other AT | CBA  PFA | 13  2 | 1.02[0.98,1.07]  1.11[1.01,1.21] | 23.7%  0.0% |
| Complications | CBA  PFA | 11  2 | 1.18[0.91,1.53]  1.13[0.21,6.09] | 0.0%  50.9% |
| Procedure duration | CBA  PFA | 12  2 | -1.07[-1.45,-0.68]  -1.51[-1.69,-1.32] | 95.6%  0.0% |
| Fluoroscopy duration | CBA  PFA | 11  2 | 0.48[0.09,0.87]  1.46[-0.80,3.72] | 95.4%  98.6% |

*Note.* RFA means Radiofrequency Ablation; CBA means Cryoballoon Ablation; PFA means Pulsed Field Ablation
